# Supplementary material for: Water sources aggregate parasites with increasing effects in more arid conditions
Source: Nat Commun. 2021 Dec 3;12:7066. doi: 10.1038/s41467-021-27352-y (PMC8642388; doi:10.1038/s41467-021-27352-y)
Supplement: Supplementary file 1 — Supplementary Information [file 41467_2021_27352_MOESM1_ESM.pdf]

# **Supplementary Information for**
## Water sources aggregate parasites, with increasing effects in more arid conditions

Titcomb, Georgia; Mantas, John Naisikie; Hulke, Jenna; Rodriguez, Ivan; Branch, Douglas; Young, Hillary

Corresponding author: Georgia Titcomb
Email: georgiatitcomb@gmail.com

**Contents**

Supplementary Information Text ........ 2
1. Camera trapping methods ........ 2
2. Herbivore dung measurements ........ 3
3. Fecal egg counts literature search ........ 3
4. Herbivore activity analyses ........ 3
5. Log-ratios of dung density ........ 4
6. Comparisons between filled pans and matrix sites ........ 4
7. Exposures across the landscape ........ 4
Fig. S1. Parasite Safari Classifications ........ 6
Fig. S2. Parasite Safari Validation ........ 7
Fig. S3. Dung drying observations ........ 8
Fig. S4. Daily Animal Activity ........ 9
Fig. S5. Activity Across a Rainfall Gradient ........ 10
Fig. S6. Parasite Density Near Water ........ 11
Fig. S7. Host-specific Dung and Parasite Density Experimental Results ........ 12
Fig. S8. Host-specific Dung and Parasite Density Observational Results ........ 13
Fig. S9. Parasite Density in Soils ........ 14
Fig. S10. Parasite Density in Soils across a Rainfall Gradient ........ 15
Fig. S11. Calculation of landscape area within 150m of water ........ 16
Table S1. Pathological Effects of Nematodes ........ 17
Table S2. Host and Nematode Checklist ........ 18
Table S3. Experimental System Camera Deployments ........ 21
Table S4. Observational System Camera Deployments ........ 22
Table S5. Dung Properties ........ 23
Table S6. Fecal Egg Counts ........ 24
Table S7. Experimental System Herbivore Activity GLMM Results ........ 26
Table S8. Observational System Herbivore Activity GLMM Results ........ 28
Table S9. Experimental hurdle GLMM results for dung density ........ 29
Table S10. Observational hurdle GLMM results for dung density ........ 30
Table S11. Experimental System Log-Ratio LMM Results ........ 31
Table S12. Post-hoc Tests for Log-Ratio LMMs ........ 32
Table S13. Observational System Log-Ratio LMM Results ........ 33
Table S14. Experimental System GLMMs for Matrix Sites ........ 34
Table S15. Experimental System Estimated Exposures ........ 36
Table S16. Observational System Estimated Exposures ........ 37
Table S15: Landscape-Level Exposure Comparisons ........ 38
SI References ........ 38

## Supplementary Information Text

### 1. Camera trapping methods

Experimental sites

*Setup:* We established cameras at both our drained and filled water pans for the duration of the experiment. We positioned cameras to capture animal movements at each water pan and performed walk tests to determine detection distances prior to deployment. All cameras had maximum detection distances between 12 and 15 meters across sites. Cameras were set to take 3 image bursts if movement or heat was sensed with minimal delay (1-5 seconds). We maintained cameras for a two-year period from August 2016 – August 2018, servicing on a monthly basis (Table S3).

*Identifications:* We uploaded photographs from all cameras to a citizen science website (https://www.zooniverse.org/projects/gtitcomb/parasite-safari) where volunteers assisted in classifying photographs by counting animals (Figure S1). Image sets were retired after 5 classifications. An animal was determined to be present if at least 3 of the 5 classifications stated its presence and counts for each activity were averaged across classifications.

*Validation:* We compared 12,571 identifications performed by the public and by study authors. Public identifications showed strong overlap with classifications performed by the study authors: 99% of species identified by the public were also identified by study authors, and 91% of species identified by authors were identified by the public (members of the public occasionally missed a second species in a photograph). Counts of animals present were also tightly correlated (Figure S2).

*Data aggregation:* The final dataset was created by calculating independent triggers: sequences of classifications that occurred within uninterrupted five-minute periods. We assumed that single-photo triggers corresponded to five seconds of animal presence. Although cameras were set to continually take images when motion was sensed, in many cases, motionless or slow-moving animals that remained within the camera frame did not create a continuous stream of photographs. Therefore, we then integrated animal counts within these triggers by multiplying the average count over the course of a trigger by the duration of the trigger. We calculated daily individual-seconds at each site for each animal by summing herbivore activity within each day that the camera was running (see below for example). We analyzed data from a total of 5856 trap nights across water pans from the three stages of the experiment (Table S3).

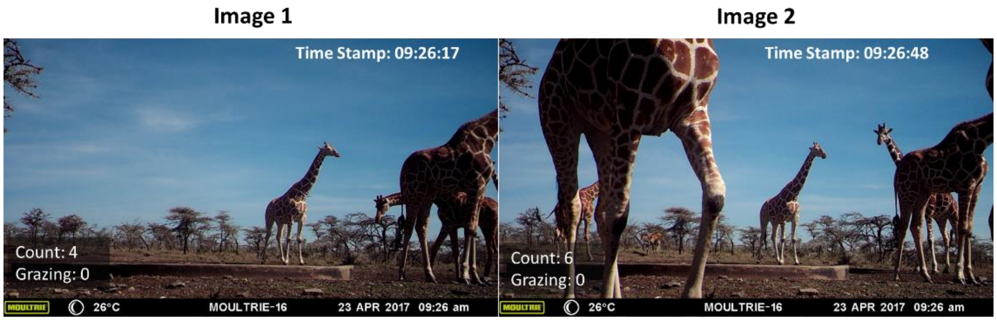


Activity Calculation
Elapsed time = 31 seconds
Mean Count = 5
Mean Grazing = 0

Activity = Elapsed time x Mean (count or grazing)
Total: 31 x 5 = 155 individual-seconds
Grazing: 31 x 0 = 0 individual-seconds

Observational sites
We also placed cameras at observational water sources and matrix sites at Mpala Research Centre to compare animal activity using the same methods as the experimental sites. From April to August 2017, we placed one camera at each water source and matrix site for at least two weeks. Of these deployments (n = 34), 12 sites ran uninterrupted for a minimum of one week at both water sources and matrix sites simultaneously (n = 24 deployments; 429 trap nights total, Table S4). Images were classified by study authors by counting animals of each species. Independent triggers (images taken within a five minute interval (6)) and animal activity (individual-seconds) were then calculated in the same manner as for the experimental sites.

## 2. Herbivore dung measurements

*Determination of 'fresh'* vs *'old'* dung:
Before the experiment began, we collected fresh dung from herbivore species included in the study (defecation was directly observed). Dung was weighed and dimensions of pellets were taken. For elephant and buffalo, a 30cm$^3$ sample was used. We placed dung in the field for two weeks and reweighed at several intervals. We found that most dung samples were very dry by day three (Figure S3); therefore, we used visible internal and external moisture content, presence of arthropods, and odor to gauge whether dung was fresh or old during our subsequent surveys. Dung quantification was applied consistently across all surveys and treatment locations by the same surveyor (J. Mantas).

*Dung volume calculation:*
To expedite field measurements, we devised a methodology to calculate the approximate volume of herbivore dung in each quadrat. For species that did not have easily countable pellets (elephants, buffalo, cattle), we counted the number of 64 cm$^2$ units of dung for each species found in each quadrat. We then multiplied this by 4 cm for elephants and 2 cm for buffalo and cattle to account for differing average depths. For all other species, we counted the number of pellets and multiplied by standard measurements from (7) to obtain the final volume.

*Physical dung density:*
We used the physical density of dung to convert parasite eggs per gram of feces to eggs per cm$^3$. We used conversions listed in Table S5 based on field measurements described above.

## 3. Fecal egg counts literature search

To compare fecal egg counts from the focal species in our study, we conducted a Web of Science literature search using the search criteria TS=((fecal AND egg AND count) AND (cow OR cattle OR elephant OR zebra OR giraffe OR buffalo OR impala)). Of the 299 results, 193 studies were selected based on the relevance of their abstracts, but only 7 contained specific FEC data from any of the focal species located in Africa. We therefore supplemented this search by investigating citations from these studies and by additional Google Scholar searches. All references are provided in Table S6. Mean fecal egg counts reported in studies from the literature search were used to construct Figure 1b in the main text.

## 4. Herbivore activity analyses

We built negative binomial GLMMs of herbivore activity (daily individual-seconds) to investigate changes throughout the course of the experiment at filled and drained pans (Table S7 and Table S8). We also compared herbivore activity at observational water sources and matrix sites at Mpala Research Centre (Table S9).

## 5. Log-ratios of dung density

To enable direct comparisons between filled and experimental pans, we calculated the log ratio for dung density ($cm^3/m^2$) at filled and experimental water pans for all parasites, all dung together, and for dung of each of the six most common species (elephants, cattle, zebra, impala, giraffe, and buffalo) (equation below). Note that zebra dung densities reflect both *Equus grevyi* and *Equus quagga*, as the dung of these two species are indistinguishable.

$$y = \log\left(\frac{Density_{filled} + 1}{Density_{experimental} + 1}\right)$$

We used linear mixed-effect models to test the effect of experiment status (pre-draining, during the experiment, and post-refill) on the log ratio of dung density. We also included outward distance and its interaction with status as fixed effects, while period (n = 10) and site (n = 5) were included as random effects. The best model of dung density log ratio was determined using backwards stepwise selection using the lmerTest package (8), and 95% confidence intervals of model coefficients were determined by bootstrapping the final model 10000 times and calculating the 95% bias-corrected confidence interval, as this method is considered robust to deviations from normal data in mixed effect models (9). If bootstrap intervals found coefficient estimates that overlapped with 0, they were dropped from the final model. Results are qualitatively similar to results presented in the main text; however, adding a nominal value (+1) to all data resulted in biased estimates for herbivore species with low dung density. Log-ratio models and post-hoc comparisons are therefore presented in Table S11 and S12 for ease of interpreting significant effects.

For our observational dataset, we also calculated the log ratios of dung and parasite density at watering holes and at matrix sites using the formula:

$$y = \log\left(\frac{Density_{waterhole} + 1}{Density_{matrix\ site} + 1}\right)$$

Log ratios of density at waterholes relative to matrix sites were analyzed using linear mixed-effect models testing the interactions between cumulative prior 30-day rainfall, mean annual precipitation, and outward distance, including random effects for site (n = 20) and period (n = 5). Best models were again determined using backwards stepwise selection using the lmerTest package (8). Final confidence intervals were again determined by bootstrapping the final model 10000 times and calculating the 95% bias-corrected percentile interval. Any non-significant interactions were dropped from the model and re-run. Model coefficients and details are reported in Table S13.

## 6. Comparisons between filled pans and matrix sites

We also created zero-inflated GLMMs to determine differences in herbivore dung and parasite aggregation between filled pans and matched matrix sites throughout the course of the experiment. Results are reported in Table S14.

## 7. Exposures across the landscape

To place our relativized results in the context of the broader landscape, we calculated the proportion of total land area found within 150m of water. Using ArcGIS Pro (v 2.5) and satellite basemap imagery, we drew polygons overlaying each water source at Mpala Research Centre and Ol Pejeta Conservancy. This included all visible water sources visible from the imagery at Mpala, and all small man-made water sources known to Ol Pejeta Conservancy (list provided by B. Gituku). Using the buffer tool, we created new polygons encompassing the 150m band surrounding each water source. We then calculated the total area of these bands and divided by the total area of each property (Fig S11). We did not include rivers or drainages in this calculation because herbivores likely aggregate differently at these areas than at the water sources in this study, due in part to the high density of surrounding trees.

We found that 1.54% and 2.61% of land fell within 150m of any provisional water source at Mpala Research Centre and Ol Pejeta Conservancy, respectively. We then used these percentages to calculate the proportion of total potential parasite exposures across the landscape that occurred within this area, assuming that matrix sites were representative of all other areas (Table S17). Specifically, we used the equation:

$$Ratio_{landscape} = \frac{Water \times Ratio_{raw}}{100 - Water}$$

Where 'Water' is the percentage of land that fell within 150m of water (1.54 or 2.61), and $Ratio_{raw}$ is the potential exposure ratio calculated per $m^2$, as reported in Figure 2F and Tables S15 and S16.

Finally, we converted this landscape exposure ratio to the percentage of parasite exposures that occurred near water compared to the rest of the landscape using:

$$\%Water = \frac{Ratio_{landscape}}{Ratio_{landscape} + 1} \times 100$$

Results are reported in Table S17.

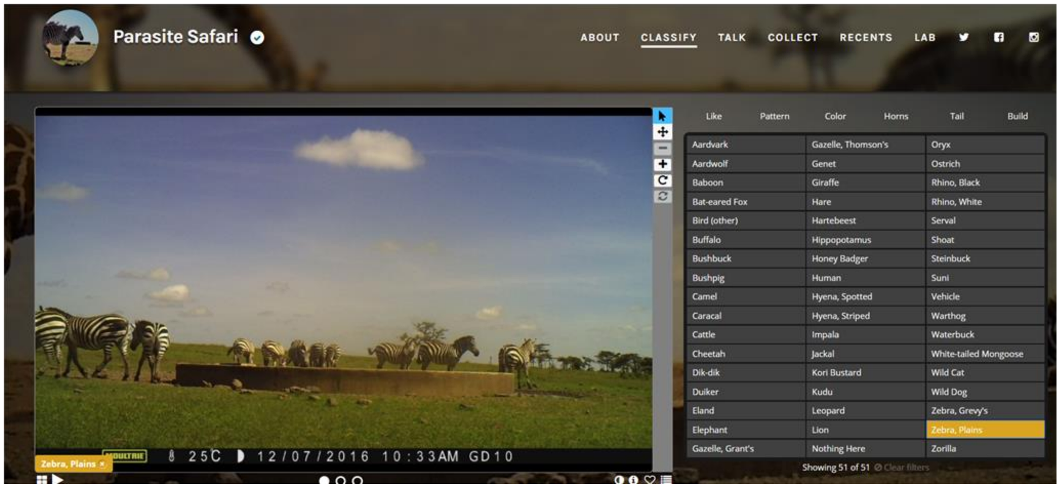


**Fig. S1. Parasite Safari Classifications**

Classification view on the Parasite Safari website used by members of the public to identify species in each image set.

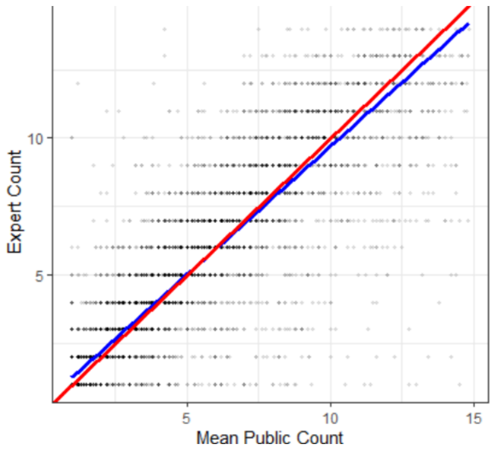


**Fig. S2. Parasite Safari Validation**

Correlation between counts of animals in each image determined by members of the public and study authors (‘expert count’) shown in blue, and the 1:1 line shown in red from 12,571 image sets. Source data are provided as a source data file.

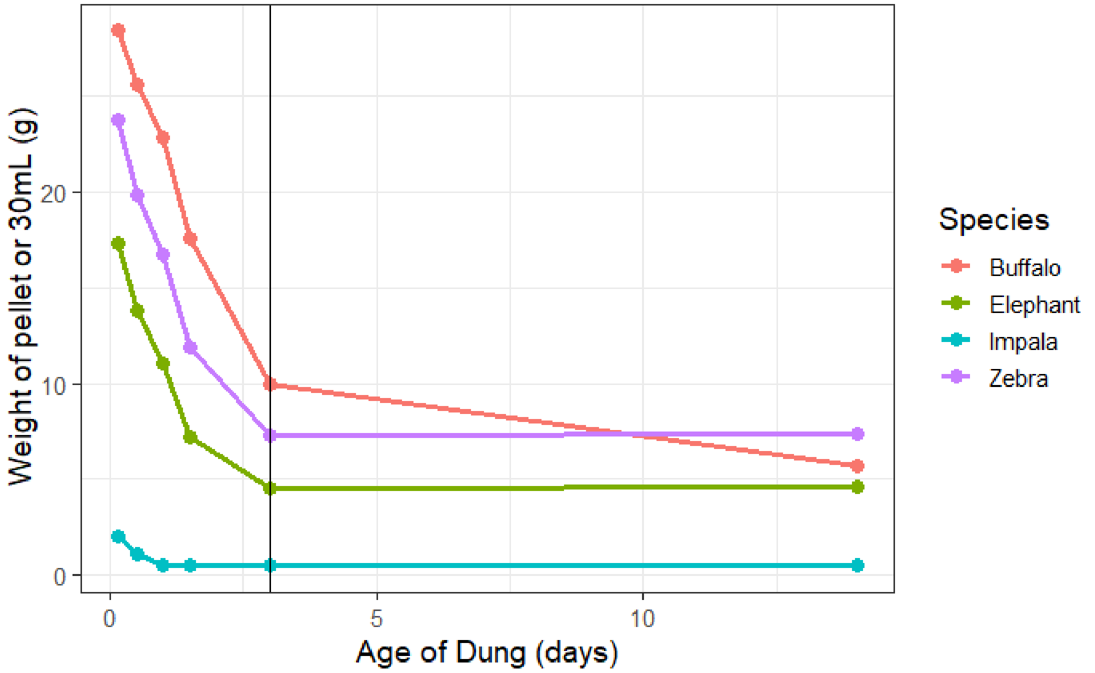


**Fig. S3. Dung drying observations**

Weight of dung pellets or 30 mL dung samples over time. Source data are provided as a source data file.

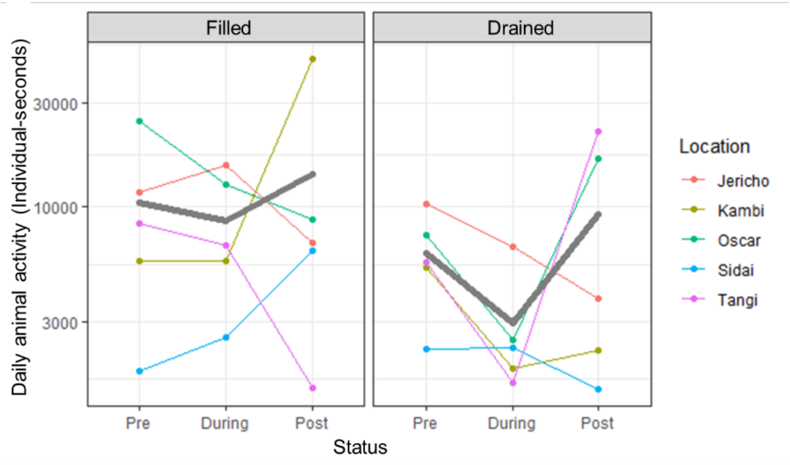


**Fig. S4. Daily Animal Activity**

Average daily animal activity (all species combined) measured by camera traps at each location throughout the experiment. Individual sites are shown in color, while the average across sites is shown in gray. Note that the plot is visualized on the $\log_{10}$ scale. Source data are provided as a source data file.

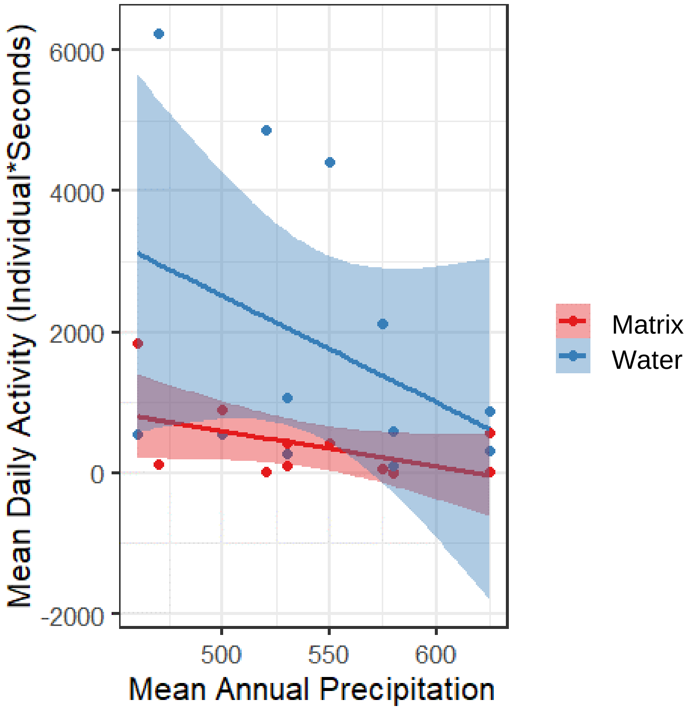


**Fig. S5. Activity Across a Rainfall Gradient**

Mean daily activity (points) for all herbivores summed together at 12 sites across Mpala Research Centre (measured by camera traps). While there was no significant interaction between MAP and water presence for any species, activity was substantially higher at water sources relative to matrix sites. Lines show linear fits to the points (±SE, n = 12 per treatment). Source data are provided as a source data file.

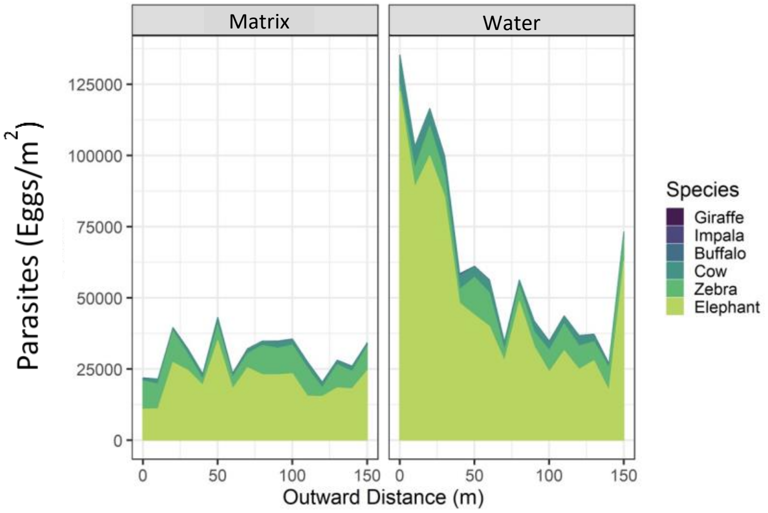


**Fig. S6. Parasite Density Near Water**

Average estimated parasite density contributed by each focal species as a function of outward distance (along transects radiating from the center of sampling sites) at water sources and paired matrix sites at Mpala. Elephants contribute the vast majority of total parasites. Source data are provided as a source data file.

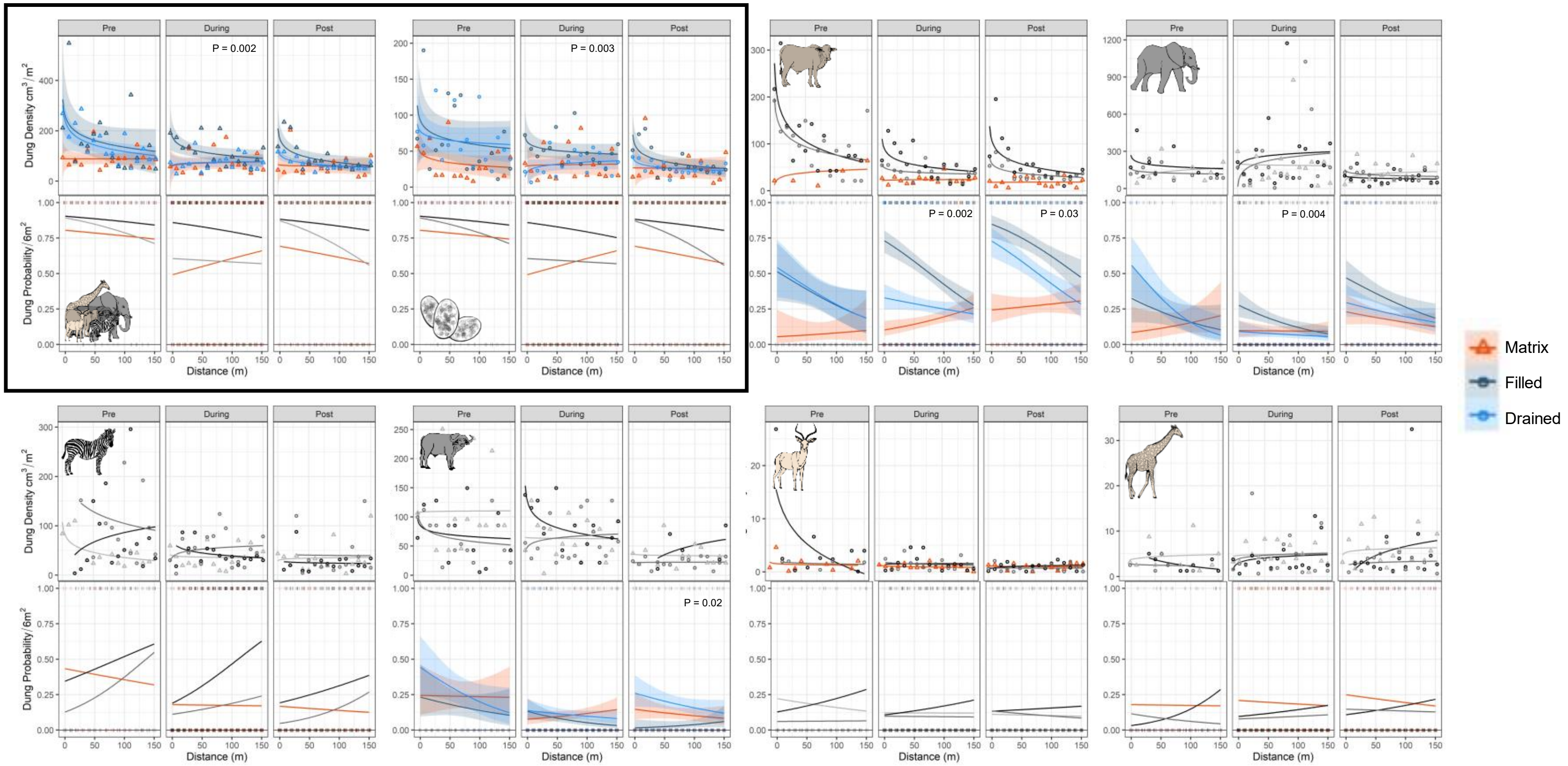


**Fig. S7. Host-specific Dung and Parasite Density Experimental Results**

Dung/parasite density and probabilities at filled pans, experimental pans, and matrix sites throughout the experiment (pre-draining, during experiment, and post-refilling). When there was a significant interaction between status and treatment, best fit lines are shaded in color with standard error bands. When filled water sources differed from matrix sites, lines are shaded in black/orange. Lines for continuous data are linear fits using the formula Density ~ log(Distance); lines for binary data are logistic fits. Points represent mean dung or parasite density at each distance, location, and experimental status (averaged across period). Source data are provided as a source data file.

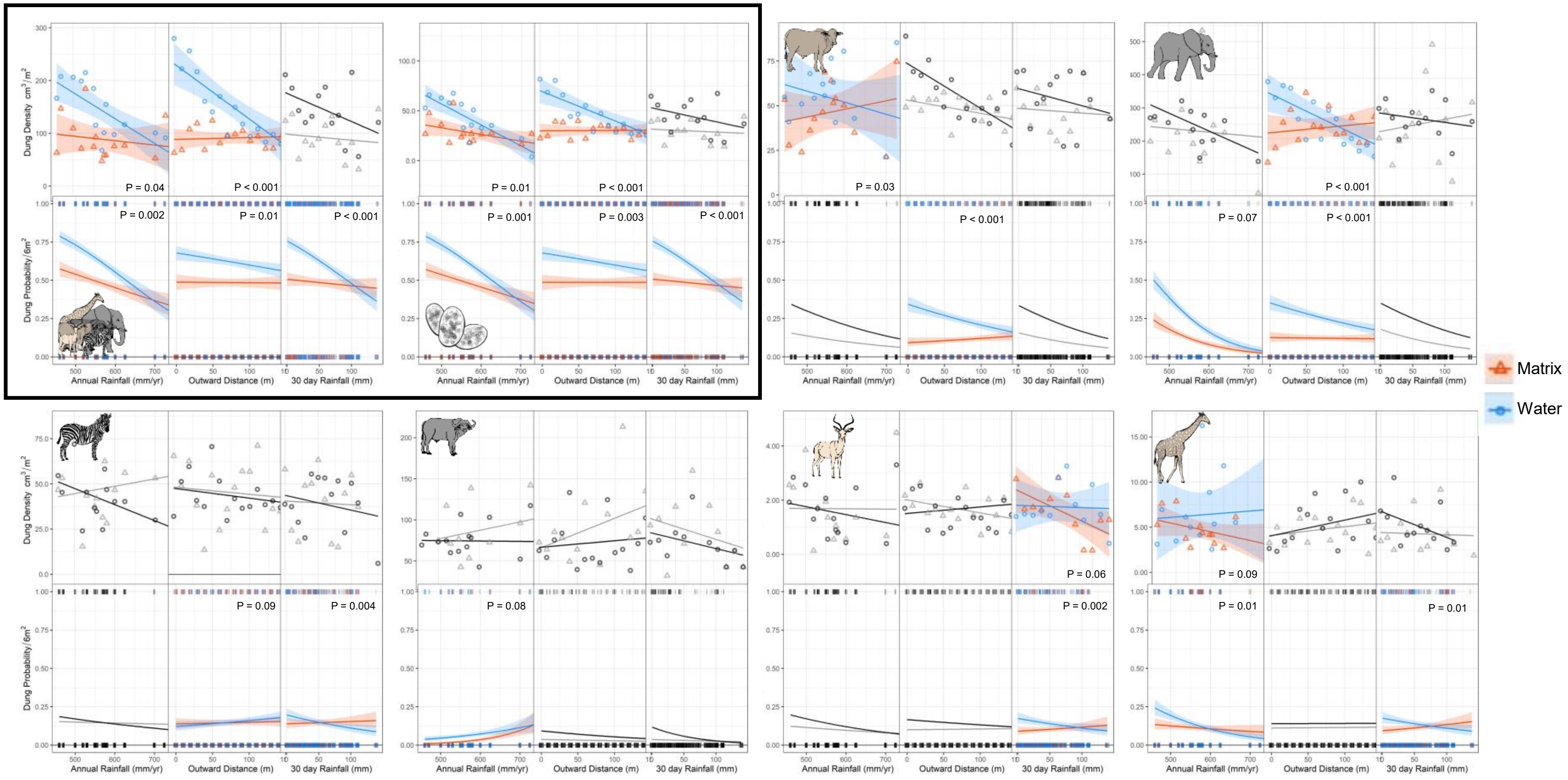


**Fig. S8. Host-specific Dung and Parasite Density Observational Results**

Dung/parasite density and probabilities at water sources and matrix sites across different rainfall contexts and outward distance from water. When there was a significant interaction between site type (water/matrix) and each covariate, best fit lines are shaded in color with standard error bands. Lines for continuous data are linear fits using the formula Density ~ log(Distance); lines for binary data are logistic fits. Points represent average dung or parasite density for each treatment and covariate value (averaged across period). Source data are provided as a source data file.

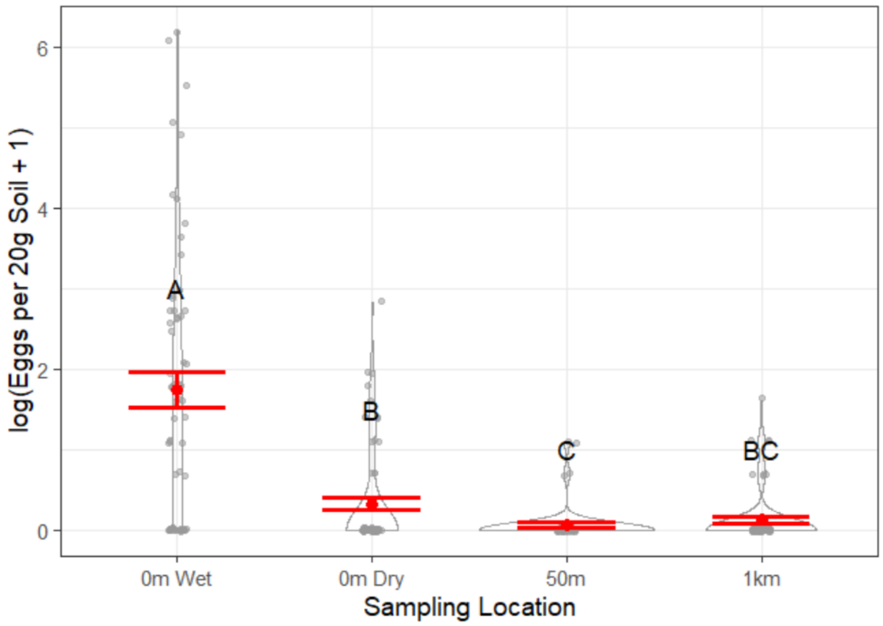


**Fig. S9. Parasite Density in Soils**

Parasite density at observational sites (Mpala) was substantially elevated in wet soils compared to matrix sites (1km). Density in dry soils next to water was also higher, but to a much lesser extent. Letters denote statistically significant groups after Tukey's correction for multiple testing. Error bars show mean ± SE (n = 61 soil egg measurements for each sampling location). Source data are provided as a source data file.

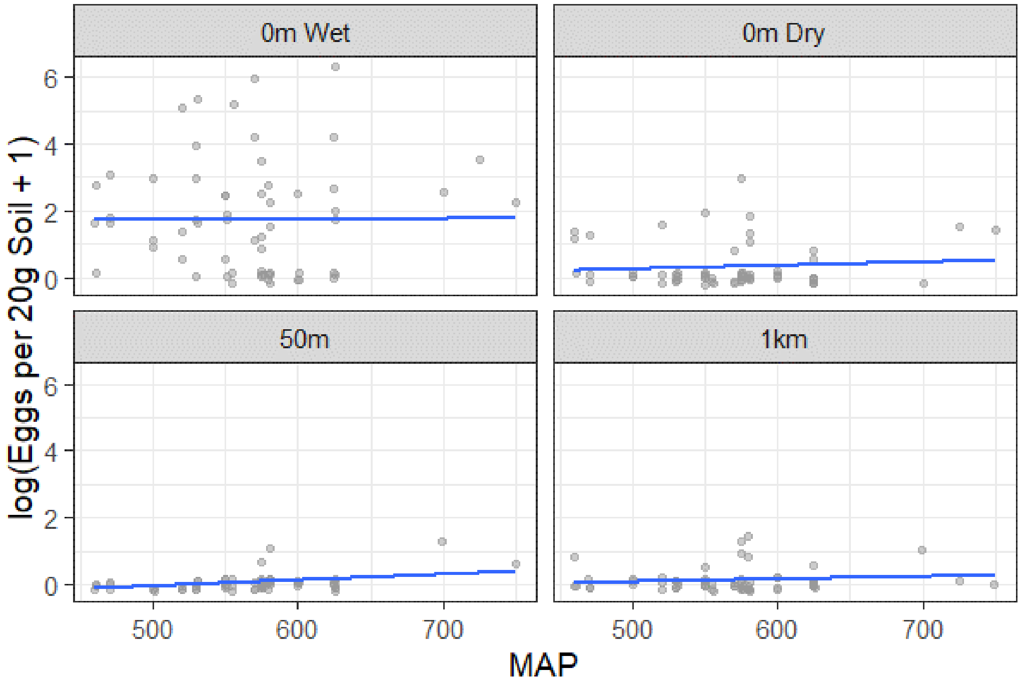


**Fig. S10. Parasite Density in Soils across a Rainfall Gradient**

Parasite density in soils increased slightly with increasing MAP across sample types. Source data are provided as a source data file.

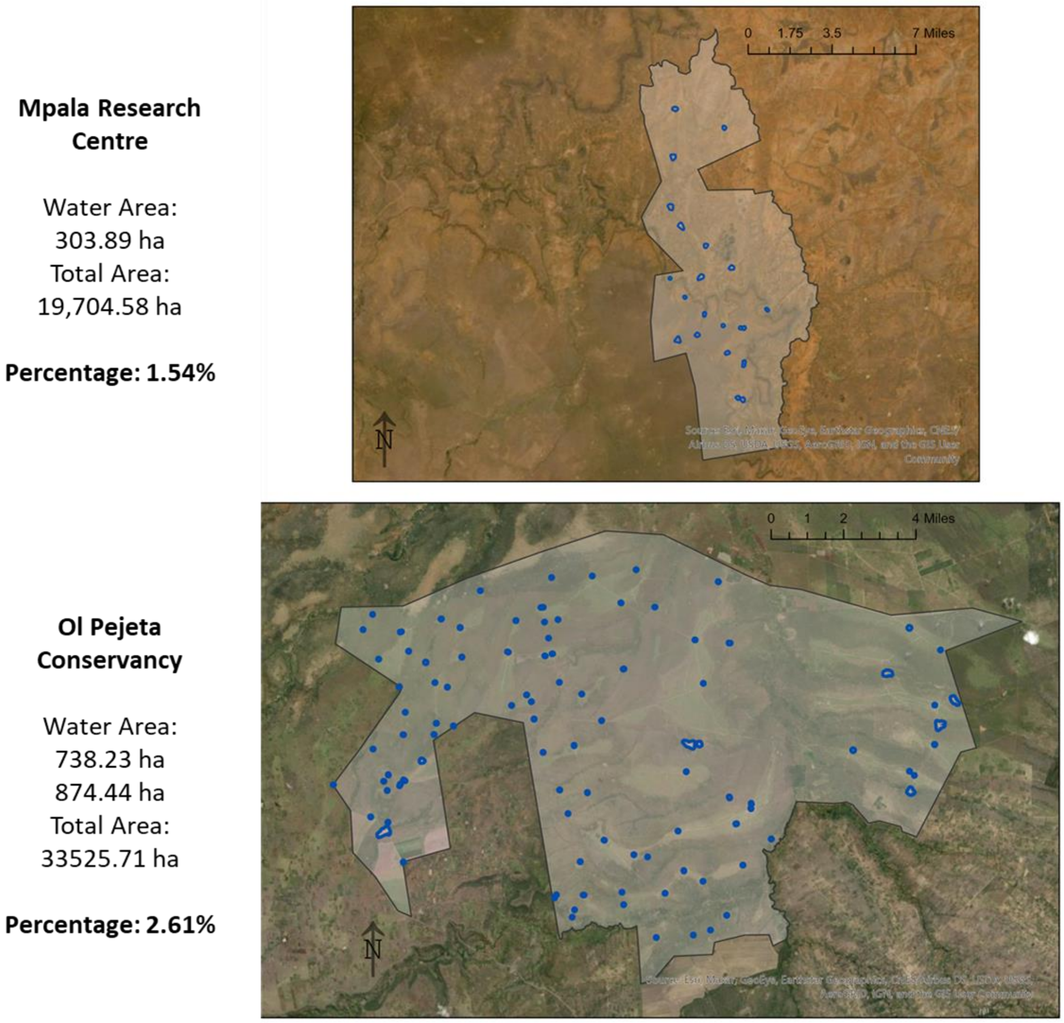


**Fig. S11. Calculation of landscape area within 150m of water**

Total area found within 150m of man-made water sources comprised only a small proportion of the total landscape at Mpala Research Centre (observational system) and Ol Pejeta Conservancy (experimental system). Satellite basemap credits: *Source*: Esri, Maxar, GeoEye, Earthstar Geographics, CNES/Airbus DS, USDA, USGS, AeroGRID, IGN, and the GIS User Community.

## Table S1. Pathological Effects of Nematodes

General pathological effects (for livestock, as listed in the Merck veterinary manual (4, 5)) of parasitic nematode groups detectable in fecal egg counts and referenced in Table S2.

| Parasite type | Order | Super Family | Family/ies | General pathology |
|---|---|---|---|---|
| Bowel Worm (BW) | Strongylida | Strongyloidea | Chabertiidae | Colon damage, diarrhea, hemorrhages with heavy infection |
| Cyathostomin (CY) | Strongylida | Strongyloidea | Strongylidae, Cyathostominae (sub) | Larvae damage intestines, reduce metabolism, and can cause colic and death in equids; milder effects in elephants |
| Large strongyle (LS) | Strongylida | Strongyloidea | Strongylidae, Strongylinae (sub) | Larvae circulate throughout the body causing variable damage, anemia, and potential ulceration in equids |
| Nodular worm (NW) | Strongylida | Strongyloidea | Oesophagostomum | Larvae penetrate intestinal walls and form nodules |
| Hookworm (HW) | Strongylida | Ancyclostomatoidea | Ancyclostomatidae | Suck host blood and can cause anemia with heavy infections |
| Thin-necked worm (TN) | Stronglyida | Molineoidea | Molineidae | Diarrhea and anorexia |
| Trichostrongyloid (TR) | Strongylida | Trichostrongyloidea | Cooperiidae<br>Dictyocaulidae<br>Haemonchidae<br>Trichostrongylidae | Larvae bury into the intestinal lining, causing damage and nutritional losses to varying degrees |
| Threadworm (TH) | Rhabditida | Tylenchina | Strongyloididae | Diarrhea and weight loss in calves, mild effects in sheep and goats |

## Table S2. Host and Nematode Checklist

Nematodes detectable from fecal egg counts, compiled using data from the Global Mammal Parasite Database (GMPD)(1), the London Museum of Natural History (2), and additional parasite checklists (3). Numbers refer to total parasite records for each host (C – cattle, E – elephant, PZ – plains zebra, GZ – Grevy's zebra, B – Buffalo, I – impala, and G – giraffe) and parasite species. Parasites of economic concern are shown in bold with additional notes, and those that have been found in rare human infections are bordered by a solid line.

| Parasite Species | C | E | PZ | GZ | B | I | G | Type | Note | Citation |
|---|---|---|---|---|---|---|---|---|---|---|
| ***Haemonchus contortus*** | 43 | | | | 4 | 8 | 2 | TR | Anemia, edema, death for sheep and goats | (10, 11) |
| ***Cooperia pectinata*** | 15 | | | | 2 | 1 | 1 | TR | Weight loss, damage to small intestine, diarrhea | (12) |
| ***Cooperia punctata*** | 40 | | | | 2 | | 1 | TR | Weight loss, diarrhea, emaciation | (13) |
| ***Cooperia oncophora*** | 79 | | | | | | 1 | TR | Mild effects; weight loss with high infections | (14) |
| ***Trichostrongylus axei*** | 41 | | | | | 1 | | TR | Weight loss, diarrhea, lethargy, death in severe infections | (15, 16) |
| ***Strongyloides papillosus*** | 25 | | | | | 5 | | TH | Weight loss, lethargy, death in sheep, goats, and cattle | (17) |
| ***Trichostrongylus colubriformis*** | 12 | | | | | 6 | | TR | Mild effects; diarrhea, weight loss, anemia | (18, 19) |
| ***Bunostomum trigonocephalum*** | 4 | | | | | 1 | | HW | Mild effects; gastroenteritis for small ruminants | (20) |
| ***Oesophagostomum columbianum*** | 3 | | | | | 2 | | NW | Damage to gut in sheep and goats | (21) |
| ***Strongylus vulgaris*** | | | 12 | 1 | | | | LS | Anorexia, colic, hemorrhage, death, severe in foals | (22) |
| ***Triodontophorus tenuicollis*** | | | 2 | 1 | | | | LS | Damage to colon, ulceration | (23) |
| ***Gaigeria pachyscelis*** | | | | | 1 | 6 | | HW | Anemia, death for sheep and goats | (24) |
| ***Ostertagia ostertagi*** | 114 | | | | | | | TR | Diarrhea, anorexia, death (calves) | (11, 25) |
| ***Haemonchus placei*** | 72 | | | | | | | TR | Similar effects as *H. contortous*; edema in calves | (26) |
| ***Oesophagostomum radiatum*** | 51 | | | | | | | NW | Anorexia, weight loss, death with very heavy infections | (27) |
| ***Bunostomum phlebotomum*** | 30 | | | | | | | HW | Anemia, weakness in sheep and cattle | (28) |
| ***Nematodirus helvetianus*** | 24 | | | | | | | TW | Reduced weight gain, fever in cattle | (29) |
| ***Mecistocirrus digitatus*** | 17 | | | | | | | TR | Anemia, reduced weight gain in calves | (30) |
| ***Teladorsagia circumcincta*** | 12 | | | | | | | TR | Reduced growth, appetite loss in sheep and lambs | (31, 32) |
| ***Chabertia ovina*** | 8 | | | | | | | BW | Weight loss (sheep and goats) | (33) |
| ***Nematodirus spathiger*** | 7 | | | | | | | TW | Anorexia, weight loss, diarrhea in sheep | (34) |
| ***Trichostrongylus vitrinus*** | 5 | | | | | | | TR | Gastrointestinal lesions in sheep | (16, 35) |
| *Cooperia curticei* | 5 | | | | | 1 | | TR | | |
| *Cooperioides hamiltoni* | 2 | | | | | 11 | | TR | | |
| *Impalaia nudicollis* | 1 | | | | | 4 | | TR | | |
| *Camelostrongylus mentulatus* | 1 | | | | | | 1 | TR | | |
| *Strongylus edentatus* | | 1 | 5 | | | | | LS | | |
| *Cylicocyclus gyalocephaloides* | | 1 | 1 | | | | | CY | | |
| *Oesophagostomum mwanzae* | | 1 | 1 | | | | | NW | | |
| *Strongylus asini* | | | 9 | 1 | | | | LS | | |
| *Cylindropharynx ornata* | | | 3 | 1 | | | | CY | | |
| *Haemonchus bedfordi* | | | | | 5 | 4 | | TR | | |
| *Cooperia fuelleborni* | | | | | 2 | 4 | | TR | | |
| *Impalaia tuberculata* | | | | | 1 | 10 | | TR | | |
| *Cooperia hungi* | | | | | 1 | 4 | | TR | | |
| *Agriostomum gorgonis* | | | | | 1 | 2 | | HW | | |
| *Haemonchus mitchelli* | | | | | | 1 | 2 | TR | | |
| *Haemonchus similis* | 29 | | | | | | | TR | | |
| *Ostertagia lyrata* | 17 | | | | | | | TR | | |
| *Ostertagia leptospicularis* | 11 | | | | | | | TR | | |
| *Trichostrongylus longispicularis* | 8 | | | | | | | TR | | |
| *Nematodirus filicollis* | 6 | | | | | | | TW | | |
| *Ostertagia kolchida* | 6 | | | | | | | TR | | |
| *Agriostomum vryburgi* | 5 | | | | | | | HW | | |
| *Oesophagostomum venulosum* | 5 | | | | | | | NW | | |
| *Ostertagia bisonis* | 5 | | | | | | | TR | | |
| *Ostertagia trifurcata* | 5 | | | | | | | TR | | |
| *Marshallagia marshalli* | 4 | | | | | | | TR | | |
| *Nematodirus oiratianus* | 4 | | | | | | | TW | | |
| *Nematodirus abnormalis* | 2 | | | | | | | TW | | |

| | | | | |
|---|---|---|---|---|
| *Nematodirus battus* | 2 | | | TW |
| *Orloffia orloffi* | 2 | | | TR |
| *Cooperia aserbaidjanica* | 1 | | | TR |
| *Cooperia sarnabada* | 1 | | | TR |
| *Haemonchus longistipes* | 1 | | | TR |
| *Hyostrongylus rubidus* | 1 | | | TR |
| *Marshallagia mongolica* | 1 | | | TR |
| *Marshallagia occidentalis* | 1 | | | TR |
| *Nematodirella longissimespiculata* | 1 | | | TR |
| *Orloffia dahurica* | 1 | | | TR |
| *Orloffia kasakhstanica* | 1 | | | TR |
| *Ostertagia buriatica* | 1 | | | TR |
| *Ostertagia crimensis* | 1 | | | TR |
| *Ostertagia gruehneri* | 1 | | | TR |
| *Ostertagia lasensis* | 1 | | | TR |
| *Spiculopteragia schulzi* | 1 | | | TR |
| *Trichostrongylus extenuatus* | 1 | | | TR |
| *Grammocephalus clathratus* | | 12 | | HW |
| *Murshidia linstowi* | | 8 | | CY |
| *Quilonia africana* | | 8 | | CY |
| *Quilonia uganda* | | 8 | | CY |
| *Murshidia africana* | | 7 | | CY |
| *Murshidia longicaudata* | | 6 | | CY |
| *Quilonia apiensis* | | 6 | | CY |
| *Khalilia sameera* | | 5 | | CY |
| *Murshidia memphisia* | | 5 | | CY |
| *Murshidia anisa* | | 4 | | CY |
| *Murshidia aziza* | | 4 | | CY |
| *Murshidia dawoodi* | | 3 | | CY |
| *Quilonia ethiopica* | | 3 | | CY |
| *Quilonia loxodontae* | | 3 | | CY |
| *Quilonia magna* | | 3 | | CY |
| *Bunostomum brevispiculum* | | 2 | | HW |
| *Bunostomum hamatum* | | 2 | | HW |
| *Murshidia brachyscelis* | | 2 | | CY |
| *Murshidia brevicapsulatus* | | 2 | | CY |
| *Murshidia omoensis* | | 2 | | CY |
| *Murshidia soudanensis* | | 2 | | CY |
| *Quilonia brevicauda* | | 2 | | CY |
| *Quilonia khalili* | | 2 | | CY |
| *Murshidia neveulemairei* | | 1 | | CY |
| *Murshidia witenbergi* | | 1 | | CY |
| *Oesophagostomum simpsoni* | | 1 | | NW |
| *Oesophagostomum yorkei* | | 1 | | NW |
| *Quilonia crenelata* | | 1 | | CY |
| *Quilonia spiculodentata* | | 1 | | CY |
| *Cylicocyclus insigne* | | | 6 | CY |
| *Cylicocyclus triramosus* | | | 6 | CY |
| *Triodontophorus serratus* | | | 5 | LS |
| *Craterostomum acuticaudatum* | | | 4 | CY |
| *Cyathostomum montgomeryi* | | | 4 | CY |
| *Poteriostomum imparidentatum* | | | 4 | CY |
| *Cylicocyclus adersi* | | | 3 | CY |
| *Cylicocyclus goldi* | | | 3 | CY |
| *Cylicostephanus minutus* | | | 3 | CY |
| *Cylindropharynx brevicauda* | | | 3 | CY |
| *Cylindropharynx longicauda* | | | 3 | CY |
| *Oesophagodontus robustus* | | | 3 | LS |
| *Poteriostomum ratzii* | | | 3 | CY |

| | | | | | | | | |
|---|---|---|---|---|---|---|---|---|
| *Strongylus equinus* | | | 3 | | | | | LS |
| *Strongylus tetracanthus* | | | 3 | | | | | LS |
| *Cyathostomum alveatum* | | | 2 | | | | | CY |
| *Cylicocyclus auriculatus* | | | 2 | | | | | CY |
| *Cylicocyclus elongatus* | | | 2 | | | | | CY |
| *Cylicostephanus longibursatus* | | | 2 | | | | | CY |
| *Cylindropharynx intermedia* | | | 2 | | | | | CY |
| *Triodontophorus burchelli* | | | 2 | | | | | LS |
| *Coronocyclus coronatus* | | | 1 | | | | | CY |
| *Coronocyclus labiatus* | | | 1 | | | | | CY |
| *Cylicocyclus leptostomum* | | | 1 | | | | | CY |
| *Cylicocyclus radiatus* | | | 1 | | | | | CY |
| *Cylicocyclus ultrajectinus* | | | 1 | | | | | CY |
| *Cylicodontophorus reineckei* | | | 1 | | | | | CY |
| *Cylicodontophorus schurmanni* | | | 1 | | | | | CY |
| *Cylicostephanus calicatus* | | | 1 | | | | | CY |
| *Cylicostephanus caragandicus* | | | 1 | | | | | CY |
| *Cylicostephanus longiconus* | | | 1 | | | | | CY |
| *Cylindropharynx dollfusi* | | | 1 | | | | | CY |
| *Gyalocephalus capitatus* | | | 1 | | | | | CY |
| *Triodontophorus brevicauda* | | | 1 | | | | | LS |
| *Ashworthius lerouxi* | | | | | 2 | | | TR |
| *Cooperioides hepaticae* | | | | | | 7 | | TR |
| *Trichostrongylus thomasi* | | | | | | 7 | | TR |
| *Cooperia yoshidai* | | | | | | 3 | | TR |
| *Haemonchus krugeri* | | | | | | 3 | | TR |
| *Trichostrongylus falculatus* | | | | | | 3 | | TR |
| *Cooperia neitzi* | | | | | | 1 | | TR |
| *Cooperia rotundispiculum* | | | | | | 1 | | TR |
| *Haemonchus lawrencei* | | | | | | 1 | | TR |
| *Haemonchus vegliai* | | | | | | 1 | | TR |
| *Oesophagostomum walkeri* | | | | | | 1 | | NW |
| *Trichostrongylus angistris* | | | | | | 1 | | TR |
| *Trichostrongylus deflexus* | | | | | | 1 | | TR |
| *Trichostrongylus instabilis* | | | | | | 1 | | TR |
| *Monodontella giraffe* | | | | | | | 6 | HW |
| *Chabertiella pesteri* | | | | | | | 1 | CY |
| *Trichostrongylus probolurus* | | | | | | | 1 | TR |

| Unidentified Species | C | E | PZ | GZ | B | I | G | Type |
|---|---|---|---|---|---|---|---|---|
| *Strongyloides sp.* | 15 | | 1 | 1 | | 2 | 2 | TH |
| *Strongylus sp.* | | | 3 | 1 | | 2 | 2 | LS |
| *Haemonchus sp.* | 18 | | | | 1 | 1 | | TR |
| *Oesophagostomum sp.* | 15 | | | | 1 | 2 | | NW |
| *Bunostomum sp.* | 11 | | | | 1 | 1 | | HW |
| *Cooperia sp.* | 24 | | | | | 1 | | TR |
| *Trichostrongylus sp.* | 21 | | | | | 2 | | TR |
| *Ostertagia sp.* | 19 | | | | | 2 | | TR |
| *Triodontophorus sp.* | | | 1 | 1 | | | | LS |
| *Nematodirus sp.* | 16 | | | | | | | TW |
| *Mecistocirrus sp.* | 2 | | | | | | | TR |
| *Chabertia sp.* | 1 | | | | | | | BW |
| *Marshallagia sp.* | 1 | | | | | | | TR |
| *Murshidia sp.* | | 6 | | | | | | CY |
| *Khalilia sp.* | | 1 | | | | | | CY |
| *Quilonia sp.* | | 1 | | | | | | CY |
| *Cylindropharynx sp.* | | | 2 | | | | | CY |
| *Cooperioides sp.* | | | | | | 1 | | TR |
| *Impalaia sp.* | | | | | | 1 | | TR |

**Table S3. Experimental System Camera Deployments**

Summary of experimental system camera trap nights at Ol Pejeta Conservancy used to assess herbivore activity. 'Drained', 'Filled', and 'Matrix' refer to each of the three experimental treatments: a pan that was drained 'During' the experimental period, a pan that remained filled throughout the experiment, and a matrix site that was located at least 1km from any water source, respectively.

| Status | Site Name | Drained | Filled | Matrix | Total |
|---|---|---|---|---|---|
| Pre | Jericho | 56 | 16 | 62 | 134 |
| | Kambi | 18 | 19 | 18 | 55 |
| | Oscar | 11 | 11 | 11 | 33 |
| | Sidai | 11 | 11 | 11 | 33 |
| | Tangi | 16 | 7 | 19 | 42 |
| During | Jericho | 446 | 283 | 386 | 1115 |
| | Kambi | 380 | 170 | 322 | 872 |
| | Oscar | 372 | 180 | 184 | 736 |
| | Sidai | 202 | 253 | 333 | 788 |
| | Tangi | 285 | 352 | 393 | 1030 |
| Post | Jericho | 56 | 102 | 109 | 267 |
| | Kambi | 48 | 9 | 33 | 90 |
| | Oscar | 46 | 87 | 32 | 165 |
| | Sidai | 27 | 112 | 93 | 232 |
| | Tangi | 48 | 120 | 96 | 264 |
| | **Grand Total** | **2022** | **1732** | **2102** | **5856** |

**Table S4. Observational System Camera Deployments**

Summary of observational system camera trap deployments at Mpala Research Centre used to assess herbivore activity across a rainfall gradient.

| Water/ Matrix | Site | Camera Model | Begin Date & Time | End Date & Time | Trap Nights |
|---|---|---|---|---|---|
| Water | 1 | Reconyx HC500 Hyperfire | 4/27/2016 | 5/18/2016 | 22 |
| Matrix | 1 | Reconyx HC500 Hyperfire | 4/27/2016 | 5/18/2016 | 22 |
| Water | 2 | Reconyx HC500 Hyperfire | 6/30/2016 | 7/19/2016 | 20 |
| Matrix | 2 | Reconyx HC500 Hyperfire | 6/30/2016 | 7/19/2016 | 20 |
| Water | 5 | Reconyx HC500 Hyperfire | 8/9/2016 | 8/22/2016 | 14 |
| Matrix | 5 | Reconyx HC500 Hyperfire | 8/9/2016 | 8/22/2016 | 14 |
| Water | 6 | Reconyx HC500 Hyperfire | 8/22/2016 | 8/30/2016 | 9 |
| Matrix | 6 | Reconyx HC500 Hyperfire | 8/9/2016 | 8/22/2016 | 14 |
| Water | 7 | Reconyx HC500 Hyperfire | 6/7/2016 | 6/30/2016 | 24 |
| Matrix | 7 | Reconyx HC500 Hyperfire | 6/7/2016 | 6/30/2016 | 24 |
| Water | 10 | Reconyx RM45 Rapidfire | 6/6/2016 | 6/16/2016 | 11 |
| Matrix | 10 | Reconyx RM45 Rapidfire | 6/6/2016 | 6/27/2016 | 22 |
| Water | 12 | Moultrie MCG-M880 | 7/2/2016 | 7/19/2016 | 18 |
| Matrix | 12 | ScoutGuard 860C | 4/4/2016 | 4/22/2016 | 19 |
| Water | 13 | Reconyx HC500 Hyperfire | 6/7/2016 | 6/30/2016 | 24 |
| Matrix | 13 | Reconyx HC500 Hyperfire | 6/7/2016 | 6/30/2016 | 24 |
| Water | 14 | Moultrie MCG-M880 | 8/22/2016 | 8/30/2016 | 9 |
| Matrix | 14 | Reconyx HC500 Hyperfire | 8/22/2016 | 8/30/2016 | 9 |
| Water | 15 | Reconyx HC500 Hyperfire | 4/26/2016 | 5/18/2016 | 23 |
| Matrix | 15 | Reconyx HC500 Hyperfire | 4/26/2016 | 5/7/2016 | 12 |
| Water | 16 | Reconyx HC500 Hyperfire | 7/19/2016 | 8/9/2016 | 22 |
| Matrix | 16 | Reconyx HC500 Hyperfire | 7/19/2016 | 8/9/2016 | 22 |
| Water | 17 | Reconyx HC500 Hyperfire | 6/30/2016 | 7/11/2016 | 12 |
| Matrix | 17 | Reconyx HC500 Hyperfire | 7/1/2016 | 7/19/2016 | 19 |

**Table S5. Dung Properties**

Species-specific dung properties used to calculate parasite density

| Measurement | Elephant | Cow | Zebra | Buffalo | Impala | Giraffe |
|---|---|---|---|---|---|---|
| Density ($g/cm^3$) | 0.58 | 0.95† | 0.79 | 0.95 | 1.66 | 1.66‡ |
| Pellet dimensions (cm) | 8 x 8 x 4 | 8 x 8 x 2 | 6 x 4 x 1.5 | 8 x 8 x 2 | 1.1 x 0.6 x 0.6 | 2.5 x 2.5 x 1.5 |
| Pellet volume ($cm^3$) | 256 | 128 | 36 | 128 | 0.396 | 9.375 |

†Density assumed to be similar to buffalo measurements

‡Density assumed to be similar to impala measurements

## Table S6. Fecal Egg Counts

Fecal egg count data and references for focal species included in our study.

| Species | N | Mean | Median | Uncertainty | Prevalence | Location | Note | Method | Ref |
|---|---|---|---|---|---|---|---|---|---|
| Buffalo | 100 | 2.07 | | | 0.3 | South Africa | Wet | MM | (36) |
| Buffalo | 100 | 4.44 | | | 0.69 | South Africa | Dry | MM | (36) |
| Buffalo | 375 | 298 | | 23 (SE) | | South Africa | Early dry | MM | (37) |
| Buffalo | 375 | 409 | | 29 (SE) | | South Africa | Late dry | MM | (37) |
| Buffalo | 60 | 349 | | 54 (SE) | 0.85 | Mpala | | MM | (38) |
| Buffalo | 40 | 274 | | | | Kenya | Dry | MM | (39) |
| Buffalo | 11 | 294 | | | | Kenya | Normal | MM | (39) |
| Buffalo | 167 | 251 | | | | South Africa | M; Fig | MM | (40) |
| Buffalo | 226 | 251 | | | | South Africa | F; Fig | MM | (40) |
| Buffalo | 78 | 1000 | | | | South Africa | Fig | MM | (41) |
| Buffalo | 448 | 800 | | | | South Africa | Fig | MM | (41) |
| Buffalo | 129 | 400 | | | | South Africa | Fig | MM | (41) |
| Buffalo | 208 | 300 | | | | South Africa | Fig | MM | (41) |
| Buffalo | 100 | 300 | | | | South Africa | Fig | MM | (41) |
| Buffalo | 103 | 94.5 | | 173 (SD) | | South Africa | Y, Positive only | MM | (42) |
| Buffalo | 283 | 120.6 | | 143 (SD) | | South Africa | A, Positive only | MM | (42) |
| Cattle | 18 | 246 | | | | Ghana | F | MM | (43) |
| Cattle | 6 | 22 | | 58 (SD) | | Tanzania | | NA | (44) |
| Cattle | 6 | 90 | | 305 (SD) | | Tanzania | | NA | (44) |
| Cattle | 8 | 85 | | 152 (SD) | | Tanzania | | NA | (44) |
| Cattle | 210 | 319 | | 62 (SE) | | Ethiopia | | MM | (45) |
| Cattle | 98 | 48 | | | 0.14 | Kenya | Y | MM | (46) |
| Cattle | 321 | 18.4 | | | 0.14 | Kenya | A | MM | (46) |
| Cattle | 349 | 296 | | 37.3 (SE) | 0.51 | Kenya | | MM | (47) |
| Cattle | 46 | 80 | | | | Tanzania | A; Fig | MM | (48) |
| Cattle | 46 | 150 | | | | Tanzania | Y; Fig | MM | (48) |
| Cattle | 46 | 300 | | | | Tanzania | J; Fig | MM | (48) |
| Cattle | 23 | 100 | | | | Tanzania | A; Fig | MM | (48) |
| Cattle | 23 | 150 | | | | Tanzania | Y; Fig | MM | (48) |
| Cattle | 23 | 200 | | | | Tanzania | J; Fig | MM | (48) |
| Cattle | 32 | 125 | | | | Tanzania | A; Fig | MM | (48) |
| Cattle | 31 | 200 | | | | Tanzania | Y; Fig | MM | (48) |
| Cattle | 31 | 300 | | | | Tanzania | J; Fig | MM | (48) |
| Cattle | 31 | 245 | 200 | 31 (SE) | 0.9 | Kenya | T | NaCl Float | (49) |
| Cattle | 423 | 180.4 | | | 0.55 | South Africa | | MM | (50) |
| Cattle | 600 | 291 | 272 | 183 (SD) | 0.7 | Rwanda | Dry (15) | MM | (51) |
| Cattle | 600 | 246 | 248 | 178 (SD) | 0.63 | Rwanda | Wet (15) | MM | (51) |
| Cattle | 57 | 229 | | | 0.84 | Kenya | C; Fig | MM | (52) |
| Cattle | 56 | 325 | | | 0.93 | Kenya | Y; Fig | MM | (52) |
| Cattle | 52 | 159 | | | 0.75 | Kenya | A; Fig | MM | (52) |
| Cattle | 64 | 150 | | | | Kenya | C | MM | (52) |
| Elephant | 187 | 1100 | | 500 (SD) | | Botswana | | MM | (53) |
| Elephant | 241 | 500 | | 600 (SD) | | Botswana | | MM | (53) |

| | | | | | | | | | |
|---|---|---|---|---|---|---|---|---|---|
| Elephant | 63 | 1409 | 1375 | | | Namibia | | MM | (54) |
| Elephant | 63 | 2204 | 2138 | | | Namibia | | MM | (54) |
| Elephant | 19 | 202 | 50 | 319 (SD) | 0.93 | Kenya | F | MM | (55) |
| Elephant | 7 | 121 | 50 | 236 (SD) | 0.93 | Kenya | M | MM | (55) |
| Elephant | 16 | 106 | 75 | 125 (SD) | 0.93 | Kenya | M | MM | (55) |
| Elephant | 35 | 320 | 200 | 419 (SD) | 0.93 | Kenya | F | MM | (55) |
| Elephant | 4 | 275 | 175 | 333 (SD) | 0.93 | Kenya | M | MM | (55) |
| Elephant | 14 | 171 | 50 | 272 (SD) | 0.93 | Kenya | M | MM | (55) |
| Elephant | 46 | 146 | 100 | 205 (SD) | 0.93 | Kenya | F | MM | (55) |
| Elephant | 8 | 200 | 100 | 276 (SD) | 0.93 | Kenya | M | MM | (55) |
| Elephant | 19 | 89 | 50 | 133 (SD) | 0.93 | Kenya | M | MM | (55) |
| Elephant | 25 | 36 | 0 | 67 (SD) | 0.93 | Kenya | F | MM | (55) |
| Elephant | 10 | 0 | 0 | 0 (SD) | 0.93 | Kenya | M | MM | (55) |
| Elephant | 22 | 23 | 0 | 46 (SD) | 0.93 | Kenya | M | MM | (55) |
| Elephant | 578 | 1694 | | 61 (SE) | 0.96 | Kenya | | MM | (56) |
| Elephant | 119 | 736 | | 84 (SE) | | Namibia | M | MM | (57) |
| Elephant | 70 | 976 | | 134 (SE) | | Namibia | F/J | MM | (57) |
| Giraffe | 21 | 77 | | | 0.33 | UK | Captive, treated | MM | (58) |
| Giraffe | 14 | 0 | 0 | | 0.06 | Kenya | Est mean | MM | (59) |
| Impala | 692 | 963 | | 39 (SE) | 0.96 | Mpala | | MM | (38) |
| Impala | 442 | 660 | | | | Kenya | Dry | MM | (39) |
| Impala | 225 | 467 | | | | Kenya | Normal | MM | (39) |
| Impala | 112 | 55 | | | | Zambia | Cool dry | MM | (60) |
| Impala | 112 | 39 | | | | Zambia | Hot dry | MM | (60) |
| Impala | 112 | 264 | | | | Zambia | Wet | MM | (60) |
| Impala | 102 | 216 | | 18 (SE) | | Uganda | Nat. Park | CF | (61) |
| Impala | 76 | 247 | | 31 (SE) | | Uganda | Ranched | CF | (61) |
| Zebra (G) | 39 | 569 | 500 | 60 (SE) | 0.82 | Kenya | T | NaCl Float | (49) |
| Zebra (G) | 15 | 1306 | | | | Kenya | J | MM | (62) |
| Zebra (G) | 47 | 1187 | | | | Kenya | J | MM | (62) |
| Zebra (G) | 145 | 1635 | | | | Kenya | A | MM | (62) |
| Zebra (G) | 15 | 1100 | | 300 (SE) | | Kenya | | | (63) |
| Zebra (P) | 76 | 317.9 | | | | Uganda | F | MM | (64) |
| Zebra (P) | 65 | 241.5 | | | | Uganda | M | MM | (64) |
| Zebra (P) | 31 | 473 | 350 | 67 (SE) | 0.61 | Kenya | T | NaCl Float | (49) |
| Zebra (P) | 5 | 2500 | 2150 | 628 (SE) | 1 | Namibia | | Krecek 1983 | (65) |
| Zebra (P) | 15 | 2100 | | 300 (SE) | | Kenya | | | (63) |
| Zebra (P) | 247 | 1600 | | 250 (SE) | | Namibia | J; Fig | MM | (66) |
| Zebra (P) | 247 | 2600 | | 200 (SE) | | Namibia | Y; Fig | MM | (66) |
| Zebra (P) | 247 | 2500 | | 100 (SE) | | Namibia | A; Fig | MM | (66) |
| Zebra (P) | 10 | 1225 | | 104 (SE) | 0.8 | Kenya | Ranched | MM | (67) |
| Zebra (P) | 10 | 1620 | | 204 (SE) | 1 | Kenya | Free Ranging | MM | (67) |

Notes: J = Juvenile, Y = Yearling, A = Adult, F = Female, M = Male
Fig: Values estimated from figures, Tab: Values calculated from table
MM = McMaster (or modified) egg float method

## Table S7. Experimental System Herbivore Activity GLMM Results

Coefficients (log-means), standard errors, and statistical results for best negative binomial GLMMs (one model per row) of herbivore total activity (T) grazing (leaf) and drinking (droplet) activity (daily individual-seconds) for the experimental system. Significant negative interactions (in blue) between status (pre, during, post) and treatment (filled, drained) indicate decreases in these behaviors at experimental pans relative to filled pans after the first period of the experiment. Significant positive interactions (in green) suggest increases after refilling relative to initial levels.

| Key: Est. ± SE<br>Z (P-value) | | (Intercept) | Drained | During | Post | During:<br>Drained | Post:<br>Drained | $R^2_{Cond}$<br>$R^2_{Mar}$ | $\sigma^2$<br>$\tau_{00\ Location}$ | Fam. |
|---|---|---|---|---|---|---|---|---|---|---|
| All | T | 9.15 ± 0.34<br>27.1 (<0.001) | -0.51 ± 0.33<br>-1.53 (0.13) | -0.24 ± 0.27<br>-0.86 (0.39) | -0.33 ± 0.29<br>-1.13 (0.26) | -0.59 ± 0.34<br>-1.71 (0.09) | 0.94 ± 0.39<br>2.44 (0.01) | 1.00<br>0.56 | 0.23<br>0.19 | NB |
| | (leaf) | 7.74 ± 0.29<br>26.54 (<0.001) | -0.46 ± 0.27<br>-1.71 (0.09) | -0.15 ± 0.22<br>-0.67 (0.5) | -0.09 ± 0.23<br>-0.39 (0.69) | -0.4 ± 0.28<br>-1.47 (0.14) | 0.85 ± 0.31<br>2.76 (0.01) | 0.02<br>0.01 | 19.50<br>0.16 | T |
| | (droplet) | 8.04 ± 0.35<br>22.92 (<0.001) | -0.67 ± 0.26<br>-2.51 (0.01) | -0.37 ± 0.21<br>-1.7 (0.09) | -0.39 ± 0.23<br>-1.66 (0.10) | -0.64 ± 0.27<br>-2.32 (0.02) | 1.45 ± 0.3<br>4.75 (<0.001) | 0.04<br>0.02 | 22.28<br>0.35 | T |
| Cattle | T | 8.05 ± 0.41<br>19.51 (<0.001) | -0.37 ± 0.45<br>-0.82 (0.41) | -0.04 ± 0.37<br>-0.11 (0.92) | 0.11 ± 0.39<br>0.27 (0.79) | -1.02 ± 0.47<br>-2.18 (0.03) | 0.19 ± 0.53<br>0.35 (0.73) | 0.01<br>0.01 | 61.86<br>0.11 | T |
| | (leaf) | 6.81 ± 0.44<br>15.57 (<0.001) | -0.31 ± 0.47<br>-0.66 (0.51) | 0.01 ± 0.38<br>0.04 (0.97) | 0.13 ± 0.41<br>0.32 (0.75) | -0.95 ± 0.48<br>-1.97 (0.05) | 0.26 ± 0.54<br>0.48 (0.63) | 0.01<br>0.01 | 66.86<br>0.14 | T |
| | (droplet) | 7.07 ± 0.44<br>15.98 (<0.001) | -0.45 ± 0.47<br>-0.96 (0.34) | -0.25 ± 0.38<br>-0.66 (0.51) | 0.11 ± 0.41<br>0.26 (0.80) | -1.12 ± 0.48<br>-2.31 (0.02) | 0.63 ± 0.54<br>1.16 (0.25) | 0.02<br>0.01 | 77.05<br>0.17 | T |
| Elephant | T | 6.61 ± 0.39<br>17.02 (<0.001) | 0.03 ± 0.39<br>0.08 (0.93) | 0.08 ± 0.33<br>0.25 (0.80) | -0.33 ± 0.36<br>-0.94 (0.35) | -1.6 ± 0.41<br>-3.91 (<0.001) | 0.92 ± 0.44<br>2.08 (0.04) | 0.02<br>0.02 | 36.66<br>0.20 | T |
| | (leaf) | 4.77 ± 0.45<br>10.72 (<0.001) | 0.18 ± 0.43<br>0.41 (0.68) | 0.18 ± 0.37<br>0.5 (0.61) | 0.03 ± 0.39<br>0.07 (0.94) | -1.24 ± 0.45<br>-2.77 (0.01) | 1.06 ± 0.48<br>2.21 (0.03) | 0.02<br>0.01 | 44.57<br>0.32 | T |
| | (droplet) | 6.05 ± 0.44<br>13.7 (<0.001) | -0.20 ± 0.41<br>-0.48 (0.63) | -0.11 ± 0.34<br>-0.32 (0.75) | -0.96 ± 0.37<br>-2.63 (0.01) | -1.58 ± 0.42<br>-3.75 (<0.001) | 1.74 ± 0.46<br>3.78 (<0.001) | 0.03<br>0.02 | 46.67<br>0.41 | T |
| Zebra | T | 7.62 ± 0.54<br>14.08 (<0.001) | -0.97 ± 0.36<br>-2.68 (0.01) | -0.39 ± 0.31<br>-1.27 (0.2) | -1.24 ± 0.33<br>-3.72 (<0.001) | -0.29 ± 0.37<br>-0.79 (0.43) | 0.66 ± 0.43<br>1.54 (0.12) | 0.05<br>0.01 | 33.26<br>0.79 | T |
| | (leaf) | 6.33 ± 0.5<br>12.74 (<0.001) | -0.9 ± 0.42<br>-2.15 (0.03) | -0.39 ± 0.35<br>-1.1 (0.27) | -0.86 ± 0.38<br>-2.25 (0.02) | 0.2 ± 0.43<br>0.46 (0.64) | 0.71 ± 0.49<br>1.44 (0.15) | 0.02<br>0.00 | 40.15<br>0.47 | T |
| | (droplet) | 6.01 ± 0.66<br>9.06 (<0.001) | -0.93 ± 0.39<br>-2.39 (0.02) | -0.41 ± 0.33<br>-1.24 (0.22) | -1.29 ± 0.36<br>-3.56 (<0.001) | -0.53 ± 0.4<br>-1.33 (0.18) | 1.00 ± 0.47<br>2.13 (0.03) | 0.05<br>0.01 | 43.16<br>1.40 | T |
| Buffalo | T | 5.82 ± 0.83<br>7.04 (<0.001) | -0.89 ± 0.77<br>-1.16 (0.25) | 0.74 ± 0.68<br>1.09 (0.28) | 0.54 ± 0.73<br>0.75 (0.46) | 0.16 ± 0.79<br>0.21 (0.84) | 2.34 ± 0.89<br>2.63 (0.01) | 1.00<br>0.18 | 0.04<br>0.94 | NB |
| | (leaf) | 4.47 ± 0.71<br>6.31 (<0.001) | -0.74 ± 0.62<br>-1.19 (0.23) | 0.34 ± 0.51<br>0.67 (0.50) | 0.46 ± 0.55<br>0.84 (0.40) | 0.50 ± 0.45<br>0.78 (0.44) | 2.06 ± 0.69<br>2.97 (0.003) | 0.03<br>0.00 | 67.26<br>1.12 | T |
| | (droplet) | 5.15 ± 0.71<br>7.21 (<0.001) | -1.37 ± 0.60<br>-2.29 (0.02) | 0.02 ± 0.48<br>0.03 (0.97) | -0.07 ± 0.52<br>-0.13 (0.90) | 0.74 ± 0.62<br>1.20 (0.23) | 2.96 ± 0.67<br>4.41 (<0.001) | 0.03<br>0.00 | 67.26<br>1.25 | T |
| Impala | T | 5.40 ± 0.92<br>5.85 (<0.001) | 0.44 ± 0.76<br>0.57 (0.57) | -0.001 ± 0.66<br>-0.001 (0.999) | -0.08 ± 0.70<br>-0.11 (0.91) | -0.95 ± 0.79<br>-1.21 (0.23) | -0.62 ± 0.87<br>-0.71 (0.48) | 1.00<br>0.03 | 0.05<br>1.86 | NB |
| | (leaf) | 4.13 ± 0.80<br>5.15 (<0.001) | -0.46 ± 0.71<br>-0.64 (0.52) | -0.12 ± 0.59<br>-0.21 (0.84) | -0.09 ± 0.63<br>-0.15 (0.88) | 0.08 ± 0.73<br>0.11 (0.91) | 0.26 ± 0.79<br>0.33 (0.74) | 0.03<br>0.00 | 66.72<br>1.31 | T |
| | (droplet) | 3.82 ± 0.89<br>4.27 (<0.001) | -0.44 ± 0.68<br>-0.65 (0.52) | -0.57 ± 0.56<br>-1.01 (0.31) | -1.00 ± 0.60<br>-1.66 (0.10) | -0.47 ± 0.70<br>-0.68 (0.50) | 0.45 ± 0.77<br>0.59 (0.56) | 0.04<br>0.00 | 82.77<br>2.20 | T |

| Key: Est. ± SE<br>Z (P-value) | | (Intercept) | Drained | During | Post | During:<br>Drained | Post:<br>Drained | $R^2_{Cond}$<br>$R^2_{Mar}$ | $\sigma^2$<br>$\tau_{00\ Location}$ | Fam. |
|---|---|---|---|---|---|---|---|---|---|---|
| | T | 5.01 ± 0.97<br>5.17 (<0.001) | 0.17 ± 1.03<br>0.16 (0.87) | -0.47 ± 0.87<br>-0.53 (0.59) | -1.39 ± 0.93<br>-1.49 (0.14) | -0.62 ± 1.05<br>-0.59 (0.56) | 0.82 ± 1.19<br>0.69 (0.49) | 1.00<br>0.11 | 0.02<br>0.87 | NB |
| Giraffe | 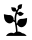 | 2.92 ± 0.79<br>3.71 (<0.001) | 0.09 ± 0.70<br>0.13 (0.89) | -0.38 ± 0.61<br>-0.62 (0.54) | -1.13 ± 0.69<br>-1.62 (0.10) | -0.37 ± 0.72<br>-0.51 (0.61) | 1.10 ± 0.84<br>1.32 (0.19) | 0.01<br>0.00 | 92.38<br>1.21 | T |
| | 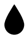 | 3.65 ± 0.81<br>4.52 (<0.001) | -0.53 ± 0.6<br>-0.87 (0.38) | -1.02 ± 0.50<br>-2.04 (0.04) | -1.69 ± 0.58<br>-2.94 (0.003) | -0.01 ± 0.63<br>-0.02 (0.98) | 1.42 ± 0.74<br>1.92 (0.05) | 0.02<br>0.00 | 92.72<br>1.93 | T |

**Table S8. Observational System Herbivore Activity GLMM Results**

Coefficients (log-means), standard errors, and statistical results for best Tweedie (Fam. = T) or negative binomial (Fam. = NB) GLMMs of herbivore total (T) or grazing (leaf) activity (daily individual-seconds) at observational water sources and matrix sites at Mpala Research Centre. Significant increases at water relative to matrix sites are shaded in blue.

| Key: | Est. ± SE<br>Z (P-value) | (Intercept)<br>Matrix,<br>MAP=4.6 | Water | MAP | Water:MAP | $R^2_{Cond}$<br>$R^2_{Mar}$ | $\sigma^2$<br>$\tau_{00\ Location}$ | Fam. |
|---|---|---|---|---|---|---|---|---|
| All | T | 4.55 ± 0.76<br>6.01 (<0.001) | 1.55 ± 0.4<br>3.9 (<0.001) | -0.01 ± 0.01<br>-1.57 (0.12) | | 0.04<br>0.02 | 48.72<br>1.05 | T |
| | 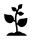 | 4.08 ± 0.78<br>5.25 (<0.001) | 0.98 ± 0.54<br>1.82 (0.07) | -0.02 ± 0.01<br>-2.43 (0.02) | | 0.02<br>0.01 | 90.22<br>0.78 | T |
| Cattle | T | 5.86 ± 1.25<br>4.69 (<0.001) | 0.78 ± 0.6<br>1.31 (0.19) | -0.02 ± 0.01<br>-1.81 (0.07) | | 0.03<br>0.01 | 152.15<br>2.50 | T |
| | 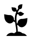 | 5.54 ± 1.12<br>4.93 (<0.001) | 0.45 ± 0.76<br>0.6 (0.55) | -0.03 ± 0.01<br>-2.11 (0.03) | | 0.02<br>0.01 | 173.23<br>1.10 | T |
| Elephant | T | 2.75 ± 1.62<br>1.7 (0.09) | 1.43 ± 0.77<br>1.87 (0.06) | -0.02 ± 0.02<br>-1.5 (0.13) | 0.02 ± 0.01<br>2.59 (0.01) | 0.18<br>0.07 | 44.91<br>6.04 | T |
| | 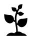 | 0.16 ± 1.4<br>0.12 (0.91) | 2.54 ± 0.57<br>4.43 (<0.001) | -0.01 ± 0.01<br>-0.68 (0.5) | | 0.10<br>0.03 | 55.70<br>4.03 | T |
| Zebra | T | 4.55 ± 0.76<br>6.01 (<0.001) | 1.55 ± 0.4<br>3.9 (<0.001) | -0.01 ± 0.01<br>-1.57 (0.12) | | 0.04<br>0.02 | 48.72<br>1.05 | T |
| | 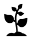 | 4.08 ± 0.78<br>5.25 (<0.001) | 0.98 ± 0.54<br>1.82 (0.07) | -0.02 ± 0.01<br>-2.43 (0.02) | | 0.02<br>0.01 | 90.22<br>0.78 | T |
| Buffalo | T | -1.08 ± 2.31<br>-0.47 (0.64) | 2.82 ± 0.79<br>3.58 (0.003) | 0.00 ± 0.02<br>0.24 (0.81) | | 0.12<br>0.02 | 83.11<br>8.85 | T |
| | 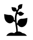 | -2.44 ± 3.75<br>-0.65 (0.52) | 2.49 ± 1.43<br>1.73 (0.08) | 0.00 ± 0.02<br>0.00 (1.00) | | 0.05<br>0.01 | 198.72<br>8.35 | T |
| Impala | T | 4.50 ± 1.48<br>3.05 (0.002) | 0.62 ± 0.86<br>0.72 (0.47) | -0.01 ± 0.01<br>-0.91 (0.36) | | 1.00<br>0.11 | 0.03<br>4.36 | NB |
| | 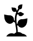 | 3.35 ± 1.12<br>2.98 (0.003) | -0.51 ± 0.68<br>-0.74 (0.46) | -0.01 ± 0.01<br>-0.76 (0.45) | | 0.02<br>0.00 | 109.55<br>1.70 | T |
| Giraffe | T | 3.08 ± 1.06<br>2.91 (0.004) | 1.86 ± 0.93<br>2 (0.05) | -0.01 ± 0.01<br>-1.6 (0.11) | | 1.00 | 0.01<br>0.00 | NB |
| | 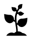 | 1.02 ± 1.2<br>0.85 (0.40) | 0.34 ± 1.05<br>0.32 (0.75) | 0.00 ± 0.01<br>-0.43 (0.67) | | 0.00 | 187.74<br>0.00 | T |

## Table S9. Experimental hurdle GLMM results for dung density

Coefficients for hurdle GLMM models of dung and parasite density for the experimental system are presented for both the conditional and zero-inflation components of the models (“Cond”, and “Zero”). Parameters signifying a decline in density at experimental pans “During” or “Post” experiment are bordered by a solid line. Significant *p*-values for two-sided *t*-tests for each coefficient (unadjusted) are given in parentheses. When dung density increased with outward distance (a pattern contrary to our expectations), parameters are bordered by a dotted line. Gray values show patterns at filled pans only. The intercept corresponds to 0m from water prior to conducting the experiment (“Pre”).

| Estimate ±SE t (P-value) | **All** | | **Elephant** | | **Cow** | | **Zebra** | | **Buffalo** | | **Impala (log)** | | **Giraffe** | | **Parasites** | |
|---|---|---|---|---|---|---|---|---|---|---|---|---|---|---|---|---|
| | Cond | Zero | Cond | Zero | Cond | Zero | Cond | Zero | Cond | Zero | Cond | Zero | Cond | Zero | Cond | Zero |
| Intercept | 5.77 | -2.92 | 5.41 | 0.01 | 5.19 | -0.88 | 3.80 | 3.00 | 4.20 | 0.83 | 0.50 | 1.63 | 0.92 | 2.91 | 36.49 | -2.97 |
| Status = Pre | ±0.87 | ±0.67 | ±0.91 | ±0.59 | ±0.63 | ±0.63 | ±0.79 | ±0.79 | ±0.57 | ±0.79 | ±0.49 | ±0.72 | ±0.47 | ±0.78 | ±5.56 | ±0.67 |
| Treat. = Filled | 6.65 | -4.34 | 5.97 | 0.01 | 8.24 | -1.39 | 4.78 | 3.80 | 7.36 | 1.05 | 1.02 | 2.25 | 1.95 | 3.72 | 6.56 | -4.44 |
| Distance = 0 | (<0.001) | (<0.001) | (<0.001) | (0.99) | (<0.001) | (0.16) | (<0.001) | (<0.001) | (<0.001) | (0.29) | 0.31 | (0.02) | (0.05) | (<0.001) | (<0.001) | (<0.001) |
| During | -0.83 | 0.36 | -0.54 | 0.35 | -1.08 | -0.81 | -0.86 | 0.30 | -0.06 | 1.14 | **-0.81** | 0.44 | 0.08 | -0.04 | -5.63 | 0.48 |
| | ±0.93 | ±0.66 | ±0.97 | ±0.60 | ±0.66 | ±0.60 | ±0.66 | ±0.69 | ±0.60 | ±0.79 | **±0.37** | ±0.69 | ±0.35 | ±0.68 | ±5.89 | ±0.67 |
| | -0.90 | 0.54 | -0.56 | 0.58 | -1.62 | -1.34 | -1.30 | 0.44 | -0.10 | 1.44 | **-2.20** | 0.64 | 0.23 | -0.05 | -0.96 | 0.73 |
| | (0.37) | (0.59) | (0.58) | (0.56) | (0.10) | (0.18) | (0.19) | (0.66) | (0.92) | (0.15) | **0.03** | (0.52) | (0.82) | (0.96) | (0.34) | (0.47) |
| Post | -0.82 | -0.07 | -1.63 | -0.66 | -1.23 | -1.68 | -1.17 | 1.04 | -0.54 | 1.93 | **-0.87** | 0.51 | 0.06 | -0.15 | -6.37 | 0.03 |
| | ±0.98 | ±0.71 | ±1.00 | ±0.63 | ±0.70 | ±0.64 | ±0.71 | ±0.73 | ±0.69 | ±0.88 | **±0.39** | ±0.73 | ±0.37 | ±0.71 | ±6.21 | ±0.71 |
| | -0.84 | -0.10 | -1.62 | -1.05 | -1.77 | -2.64 | -1.64 | 1.42 | -0.79 | 2.20 | **-2.22** | 0.69 | 0.16 | -0.21 | -1.03 | 0.04 |
| | (0.40) | (0.92) | (0.11) | (0.29) | (0.08) | (0.01) | (0.10) | (0.16) | (0.43) | (0.03) | **0.03** | (0.49) | (0.87) | (0.84) | (0.31) | (0.97) |
| Drained | 0.27 | 0.52 | -0.56 | -0.38 | -0.38 | -0.06 | 0.63 | **0.85** | -0.02 | -0.67 | -0.13 | **1.44** | -0.09 | 0.57 | 0.81 | 0.49 |
| | ±0.29 | ±0.46 | ±0.57 | ±0.39 | ±0.33 | ±0.35 | ±0.37 | **±0.36** | ±0.38 | ±0.41 | ±0.55 | **±0.58** | ±0.37 | ±0.57 | ±2.17 | ±0.45 |
| | 0.94 | 1.13 | -0.99 | -0.98 | -1.14 | -0.18 | 1.72 | **2.34** | -0.06 | -1.61 | -0.25 | **2.47** | -0.24 | 0.99 | 0.37 | 1.10 |
| | (0.35) | (0.26) | (0.32) | (0.33) | (0.25) | (0.86) | (0.09) | **(0.02)** | (0.95) | (0.11) | 0.81 | **(0.01)** | (0.81) | (0.32) | (0.71) | (0.27) |
| Outward Distance | **-0.30** | **0.22** | 0.01 | **0.37** | **-0.18** | **0.41** | -0.06 | **-0.73** | -0.07 | **0.25** | -0.03 | **-0.17** | 0.09 | **-0.23** | **-1.28** | **0.24** |
| | **±0.04** | **±0.07** | ±0.07 | **±0.05** | **±0.04** | **±0.05** | ±0.12 | **±0.09** | ±0.06 | **±0.06** | ±0.09 | **±0.08** | ±0.07 | **±0.10** | **±0.30** | **±0.06** |
| | **-7.55** | **3.31** | 0.08 | **7.17** | **-5.13** | **7.76** | -0.50 | **-8.05** | -1.16 | **3.93** | -0.37 | **-2.12** | 1.19 | **-2.41** | **-4.34** | **3.92** |
| | **(<0.001)** | **(0.001)** | (0.94) | **(<0.001)** | **(<0.001)** | **(<0.001)** | (0.62) | **(<0.001)** | (0.25) | **(<0.001)** | 0.71 | **(0.03)** | (0.23) | **(0.02)** | **(<0.001)** | **(<0.001)** |
| During: Drained | **-1.01** | 0.62 | 0.17 | **1.33** | -0.05 | **1.20** | -0.41 | 0.58 | -0.52 | 0.19 | 0.37 | -0.85 | 0.08 | -0.14 | **-7.09** | 0.64 |
| | **±0.33** | ±0.50 | ±0.69 | **±0.46** | ±0.36 | **±0.39** | ±0.42 | ±0.41 | ±0.47 | ±0.49 | ±0.59 | ±0.63 | ±0.39 | ±0.62 | **±2.42** | ±0.48 |
| | **-3.09** | 1.23 | 0.25 | **2.88** | -0.14 | **3.09** | -0.97 | 1.41 | -1.10 | 0.39 | 0.62 | -1.36 | 0.21 | -0.23 | **-2.93** | 1.32 |
| | **(0.002)** | (0.22) | (0.81) | **(0.004)** | (0.89) | **(0.002)** | (0.33) | (0.16) | (0.27) | (0.70) | 0.53 | (0.17) | (0.83) | (0.82) | **(0.003)** | (0.19) |
| Post: Drained | -0.66 | 0.28 | 1.13 | **0.94** | -0.03 | **0.90** | -0.57 | 0.33 | -0.91 | **-1.40** | -0.06 | -1.00 | -0.10 | -0.45 | -4.53 | 0.29 |
| | ±0.34 | ±0.54 | ±0.65 | **±0.45** | ±0.36 | **±0.41** | ±0.48 | ±0.46 | ±0.59 | **±0.58** | ±0.62 | ±0.66 | ±0.40 | ±0.64 | ±2.53 | ±0.52 |
| | -1.91 | 0.52 | 1.74 | **2.09** | -0.10 | **2.17** | -1.19 | 0.73 | -1.52 | **-2.40** | -0.09 | -1.52 | -0.26 | -0.71 | -1.79 | 0.56 |
| | (0.06) | (0.60) | (0.08) | **(0.04)** | (0.92) | **(0.03)** | (0.23) | (0.47) | (0.13) | **(0.017)** | 0.92 | (0.13) | (0.80) | (0.48) | (0.07) | (0.57) |
| $\sigma^2_{Site}$ | 0.04 | 0.04 | 0.00 | 0.08 | 0.06 | 0.27 | 0.01 | 0.46 | 0.09 | 0.40 | 0.00 | 0.10 | 0.02 | 0.26 | 3.69 | 0.04 |
| $\sigma^2_{Period}$ | 0.68 | 0.24 | 0.59 | 0.22 | 0.31 | 0.24 | 0.31 | 0.33 | 0.16 | 0.40 | 0.03 | 0.29 | 0.05 | 0.26 | 26.60 | 0.25 |
| $\sigma^2_{Residual}$ | 2.78 | 2.78 | 2.85 | 2.85 | 1.48 | 1.48 | 1.87 | 1.87 | 1.11 | 1.11 | 1.60 | 1.60 | 0.34 | 0.34 | 156.85 | 156.85 |
| $R^2_M$ | 0.102 | | 0.084 | | 0.111 | | 0.084 | | 0.155 | | 0.078 | | 0.045 | | 0.084 | |
| $R^2_C$ | 0.286 | | 0.209 | | 0.289 | | 0.218 | | 0.309 | | 0.158 | | 0.201 | | 0.232 | |
| N = 1440 | | | | | | | | | | | | | | | | |

## Table S10. Observational hurdle GLMM results for dung density

Coefficients for hurdle GLMM models of dung and parasite density for the observational system are presented for the conditional and zero-inflation model (“Cond”, and “Zero”). Parameters signifying a negative relationship between density and each covariate are shaded in blue (as hypothesized), while a positive relationship is shaded in orange (contrary to expectations). Significant *p*-values for two-sided *t*-tests for each coefficient (unadjusted) are given in parentheses. Gray values show patterns at matrix sites only. The intercept corresponds to dung and parasite density at matrix sites when distance and prior rainfall are zero and MAP is the lowest level observed (450 mm/yr).

| Estimate<br>±SE<br>t (P-value) | All | | Elephant | | Cow | | Zebra | | Buffalo | | Impala | | Giraffe | | Parasites | |
|---|---|---|---|---|---|---|---|---|---|---|---|---|---|---|---|---|
| | Cond | Zero | Cond | Zero | Cond | Zero | Cond | Zero | Cond | Zero | Cond | Zero | Cond | Zero | Cond | Zero |
| (Intercept)<br>MAP = 4.5<br>Dist = 0<br>Rain = 0 | 3.86<br>±0.49<br>7.85<br>(<0.001) | -0.88<br>±0.40<br>-2.22<br>(0.027) | 4.59<br>±0.60<br>7.62<br>(<0.001) | 0.63<br>±0.58<br>1.09<br>(0.28) | 3.32<br>±0.37<br>8.86<br>(<0.001) | 1.89<br>±0.58<br>3.23<br>(<0.001) | 3.51<br>±0.38<br>9.20<br>(<0.001) | 1.52<br>±0.53<br>2.90<br>(<0.001) | 4.17<br>±0.50<br>8.31<br>(<0.001) | 4.22<br>±0.93<br>4.55<br>(<0.001) | 1.18<br>±0.26<br>4.59<br>(0.00) | 2.22<br>±0.51<br>4.36<br>(<0.001) | 1.51<br>±0.19<br>8.09<br>(<0.001) | 1.85<br>±0.42<br>4.42<br>(<0.001) | 46.09<br>±8.03<br>5.74<br>(<0.001) | -2.72<br>±0.74<br>-3.69<br>(<0.001) |
| MAP | -0.33<br>±0.20<br>-1.68<br>(0.092) | **0.45<br>±0.12<br>3.83<br>(<0.001)** | -0.22<br>±0.29<br>-0.74<br>(0.46) | **0.95<br>±0.31<br>3.08<br>(0.002)** | 0.20<br>±0.14<br>1.42<br>(0.16) | **0.72<br>±0.24<br>2.98<br>(0.003)** | -0.01<br>±0.15<br>-0.05<br>(0.96) | 0.32<br>±0.25<br>1.27<br>(0.20) | -0.04<br>±0.18<br>-0.20<br>(0.84) | **-1.19<br>±0.28<br>-4.24<br>(<0.001)** | -0.17<br>±0.10<br>-1.67<br>(0.10) | 0.00<br>±0.23<br>0.02<br>(0.98) | -0.17<br>±0.10<br>-1.61<br>(0.11) | 0.36<br>±0.23<br>1.54<br>(0.12) | **-4.04<br>±1.31<br>-3.09<br>(0.002)** | **0.43<br>±0.11<br>3.78<br>(<0.001)** |
| Water | **3.14<br>±0.43<br>7.38<br>(<0.001)** | **-2.43<br>±0.37<br>-6.61<br>(<0.001)** | **2.33<br>±0.67<br>3.48<br>(0.001)** | **-2.70<br>±0.44<br>-6.19<br>(<0.001)** | **1.03<br>±0.40<br>2.60<br>(0.009)** | **-3.03<br>±0.48<br>-6.31<br>(<0.001)** | -0.01<br>±0.56<br>-0.03<br>(0.98) | -0.05<br>±0.47<br>-0.11<br>(0.91) | 0.11<br>±0.54<br>0.20<br>(0.84) | **-1.73<br>±0.62<br>-2.80<br>(0.005)** | -0.46<br>±0.37<br>-1.23<br>(0.22) | **-2.15<br>±0.74<br>-2.92<br>(0.003)** | -0.29<br>±0.27<br>-1.06<br>(0.29) | **-0.99<br>±0.47<br>-2.09<br>(0.036)** | **34.12<br>±8.06<br>4.23<br>(<0.001)** | **-4.73<br>±0.85<br>-5.58<br>(<0.001)** |
| Distance | 0.06<br>±0.07<br>0.91<br>(0.36) | 0.01<br>±0.05<br>0.12<br>(0.91) | 0.24<br>±0.12<br>1.93<br>(0.053) | 0.01<br>±0.07<br>0.11<br>(0.91) | -0.03<br>±0.07<br>-0.37<br>(0.71) | **-0.18<br>±0.08<br>-2.15<br>(0.032)** | -0.09<br>±0.07<br>-1.23<br>(0.22) | -0.03<br>±0.06<br>-0.39<br>(0.70) | 0.08<br>±0.10<br>0.86<br>(0.39) | 0.16<br>±0.11<br>1.38<br>(0.17) | -0.06<br>±0.05<br>-1.18<br>(0.24) | -0.09<br>±0.10<br>-0.92<br>(0.36) | 0.04<br>±0.04<br>1.04<br>(0.30) | 0.01<br>±0.07<br>0.20<br>(0.84) | 0.53<br>±0.48<br>1.10<br>(0.27) | 0.00<br>±0.05<br>-0.05<br>(0.96) |
| Rain | **-1.41<br>±0.27<br>-5.21<br>(<0.001)** | **0.74<br>±0.21<br>3.54<br>(<0.001)** | 0.38<br>±0.44<br>0.86<br>(0.39) | **1.59<br>±0.28<br>5.63<br>(<0.001)** | 0.00<br>±0.27<br>0.01<br>(0.99) | **1.09<br>±0.31<br>3.48<br>(0.001)** | -0.10<br>±0.29<br>-0.36<br>(0.72) | **0.60<br>±0.27<br>2.25<br>(0.02)** | -0.52<br>±0.27<br>-1.93<br>(0.054) | **1.64<br>±0.35<br>4.73<br>(<0.001)** | **-0.35<br>±0.16<br>-2.24<br>(0.025)** | -0.55<br>±0.40<br>-1.37<br>(0.17) | 0.00<br>±0.13<br>-0.01<br>(0.99) | -0.23<br>±0.27<br>-0.87<br>(0.38) | **-9.70<br>±1.93<br>-5.04<br>(<0.001)** | **0.80<br>±0.20<br>4.12<br>(<0.001)** |
| MAP:<br>Water | **-0.38<br>±0.19<br>-2.00<br>(0.045)** | **0.45<br>±0.15<br>3.04<br>(0.002)** | 0.10<br>±0.36<br>0.29<br>(0.77) | 0.41<br>±0.22<br>1.84<br>(0.065) | **-0.36<br>±0.17<br>-2.13<br>(0.034)** | 0.16<br>±0.21<br>0.76<br>(0.45) | -0.11<br>±0.22<br>-0.52<br>(0.61) | 0.21<br>±0.19<br>1.12<br>(0.26) | -0.23<br>±0.20<br>-1.15<br>(0.25) | 0.41<br>±0.24<br>1.74<br>(0.08) | 0.18<br>±0.18<br>0.98<br>(0.33) | 0.45<br>±0.37<br>1.21<br>(0.23) | 0.22<br>±0.13<br>1.70<br>(0.09) | **0.56<br>±0.22<br>2.52<br>(0.012)** | **-3.01<br>±1.36<br>-2.20<br>(0.028)** | **0.47<br>±0.14<br>3.40<br>(0.001)** |
| Distance:<br>Water | **-0.49<br>±0.09<br>-5.68<br>(<0.001)** | **0.18<br>±0.07<br>2.48<br>(0.013)** | **-0.50<br>±0.14<br>-3.55<br>(<0.001)** | **0.31<br>±0.09<br>3.56<br>(<0.001)** | -0.12<br>±0.08<br>-1.52<br>(0.13) | **0.44<br>±0.10<br>4.58<br>(<0.001)** | 0.01<br>±0.11<br>0.05<br>(0.96) | -0.17<br>±0.10<br>-1.70<br>(0.090) | -0.06<br>±0.12<br>-0.52<br>(0.60) | 0.10<br>±0.14<br>0.70<br>(0.48) | 0.01<br>±0.07<br>0.14<br>(0.89) | 0.12<br>±0.14<br>0.89<br>(0.38) | 0.04<br>±0.06<br>0.80<br>(0.42) | -0.05<br>±0.10<br>-0.56<br>(0.58) | **-3.15<br>±0.62<br>-5.08<br>(<0.001)** | **0.21<br>±0.07<br>3.01<br>(0.003)** |
| Rain:<br>Water | -0.05<br>±0.32<br>-0.16<br>(0.87) | **1.23<br>±0.25<br>4.99<br>(<0.001)** | -0.72<br>±0.52<br>-1.37<br>(0.17) | 0.15<br>±0.32<br>0.46<br>(0.64) | -0.33<br>±0.31<br>-1.07<br>(0.28) | 0.27<br>±0.36<br>0.76<br>(0.45) | 0.15<br>±0.38<br>0.39<br>(0.69) | **0.95<br>±0.33<br>2.86<br>(0.004)** | | | 0.41<br>±0.22<br>1.88<br>(0.060) | **1.58<br>±0.52<br>3.07<br>(0.002)** | -0.19<br>±0.18<br>-1.04<br>(0.30) | **0.87<br>±0.33<br>2.63<br>(0.012)** | -0.37<br>±2.32<br>-0.16<br>(0.87) | **1.18<br>±0.23<br>5.06<br>(<0.001)** |
| $\sigma_{Site}$<br>$\sigma_{Period}$<br>$\sigma_{Residual}$ | 0.43<br>0.69<br>2.08 | 0.19<br>0.67<br>2.08 | 0.09<br>0.33<br>1.91 | 0.74<br>0.73<br>1.91 | 0.10<br>0.25<br>0.91 | 0.50<br>0.80<br>0.91 | 0.12<br>0.13<br>1.18 | 0.65<br>0.69<br>1.18 | 0.00<br>0.30<br>0.95 | 0.64<br>1.49<br>0.95 | 0.20<br>0.00<br>0.56 | 0.35<br>0.26<br>0.56 | 0.00<br>0.00<br>0.55 | 0.48<br>0.25<br>0.55 | 2.63<br>4.79<br>15.43 | 0.20<br>0.68<br>15.43 |
| $R^2_M$<br>$R^2_C$ | 0.128<br>0.244 | | 0.032<br>0.062 | | 0.042<br>0.119 | | 0.010<br>0.031 | | | | | | | | 0.128<br>0.226 | |
| N = 2816 | | | | | | | | | | | | | | | | |

## Table S11. Experimental System Log-Ratio LMM Results

Best models and coefficients for log ratios of dung and parasite densities at filled water pans versus experimental pans. Parameters indicating increased dung density at filled pans relative to drained pans are highlighted in green, while those indicating decreased density are highlighted in orange. Distance is scaled such that a single unit increase in distance corresponds with 100m outward from water. The intercept corresponds to 0m from water prior to conducting the experiment (“Pre”). Both 95% profile and bias-corrected bootstrap confidence intervals (from 10,000 bootstraps) are provided for each coefficient.

| Species | Intercept (0m, Pre) | During | Post | Distance | Distance: During | Distance: Post | $\sigma^2$<br>$\sigma^2_{site}$<br>$\sigma^2_{period}$ |
|---|---|---|---|---|---|---|---|
| **All** | -0.15<br>-0.27<br>-1.23, 0.93<br>-1.21, 0.96<br>0.786 | 1.97<br>3.31<br>0.80, 3.14<br>0.74, 3.12<br>**0.002** | 0.73<br>1.16<br>-0.50, 1.96<br>-0.44, 1.89<br>0.25 | 0.24<br>0.41<br>-0.89, 1.36<br>-0.92, 1.35<br>0.682 | -1.18<br>-1.87<br>-2.41, 0.06<br>-2.41, 0.088<br>0.062 | -0.19<br>-0.28<br>-1.49, 1.11<br>-1.44, 1.11<br>0.777 | 5.62<br>0.03<br>0.04 |
| **Cow** | -0.03<br>-0.06<br>-0.99 – 0.93<br>-1.14, 1.01<br>0.95 | 1.87<br>3.83<br>0.91, 2.84<br>0.76, 3.04<br>**<0.001** | 0.96<br>0.52<br>-0.06, 1.97<br>-0.19, 2.21<br>0.064 | 0.046<br>0.09<br>-0.95, 1.04<br>-1.14, 1.29<br>0.93 | -1.27<br>-2.28<br>-2.36, -0.17<br>-2.59, -0.001<br>**0.023** | -0.30<br>-0.52<br>-1.46, 0.85<br>-1.71, 1.00<br>0.60 | 4.45<br>0.17<br>0.00 |
| **Elephant** | -0.035<br>-0.086<br>-0.89, 0.82<br>-0.86, 0.35<br>0.932 | 0.66<br>2.01<br>-0.066, 1.38<br>**0.045, 1.31**<br>0.078 | 0.51<br>1.47<br>-0.26, 1.27<br>-0.17, 1.19<br>0.18 | | | | 5.50<br>0.28<br>0.02 |
| **Zebra** | 0.53<br>7.72<br>0.38, 0.68<br>0.39, 0.67<br>**<0.001** | | | | | | 3.39<br>0.00<br>0.00 |
| **Impala** | 0.19<br>3.47<br>0.072, 0.311<br>0.062, 0.346<br>**0.006** | -0.16<br>-2.87<br>-0.284, -0.038<br>-0.315, -0.026<br>**0.02** | -0.15<br>-2.61<br>-0.283, -0.024<br>-0.313, -0.015<br>**0.03** | | | | 0.18<br>0.00<br>0.00 |
| **Buffalo** | -0.22<br>-1.32<br>-0.60, 0.16<br>**-0.33, -0.12**<br>0.23 | | | | | | 2.20<br>0.10<br>0.05 |
| **Giraffe** | -0.028<br>-0.50<br>-0.14, 0.085<br>-0.11, 0.052<br>0.62 | | | 0.11<br>2.00<br>0.002, 0.218<br>0.018, 0.207<br>**0.046** | | | 0.46<br>0.00<br>0.00 |
| **All Parasites** | 0.50<br>0.80<br>-0.73, 1.72<br>-0.66, 1.69<br>0.45 | 2.12<br>3.10<br>0.78, 3.46<br>0.82, 3.38<br>**0.01** | 0.99<br>1.37<br>-0.42, 2.40<br>-0.33, 2.30<br>0.20 | | | | 27.60<br>0.00<br>0.05 |

| **Legend:** | Estimate<br>t-value<br>95% Profile interval<br>95% Bootstrap interval<br>Pr(>\|t\|) |
|---|---|

### Table S12. Post-hoc Tests for Log-Ratio LMMs

Post-hoc tests for models in Table S9 using the Tukey correction for multiple comparisons (two-sided p-values; Z-test). For best models that included an interaction between distance and experiment status, estimates reflect ratios at 0m outward distance. The converted ratios are shown in the far-right column of the table.

| Species | Null Hypothesis | Estimate | SE | Z | P | 95% CI: Log Ratio Lower | Upper | Ratio Estimate |
|---|---|---|---|---|---|---|---|---|
| All | During - Pre == 0 | 1.973 | 0.597 | 3.307 | 0.003 ** | 0.584 | 3.361 | **7.189** |
| | Post - Pre == 0 | 0.730 | 0.629 | 1.161 | 0.469 | -0.734 | 2.193 | 2.075 |
| | Post - During == 0 | -1.243 | 0.398 | -3.125 | 0.005 ** | -2.168 | -0.317 | **0.289** |
| Cow | During - Pre == 0 | 0.924 | 0.258 | 3.578 | <0.001 *** | 0.323 | 1.525 | **2.519** |
| | Post - Pre == 0 | 0.730 | 0.272 | 2.682 | 0.02 * | 0.097 | 1.364 | **2.075** |
| | Post - During == 0 | -0.194 | 0.172 | -1.127 | 0.490 | -0.595 | 0.217 | 0.824 |
| Elephant | During - Pre == 0 | 0.659 | 0.328 | 2.01 | 0.106 | -0.104 | 1.423 | 1.933 |
| | Post - Pre == 0 | 0.509 | 0.346 | 1.47 | 0.298 | -0.296 | 1.314 | 1.664 |
| | Post - During == 0 | -0.150 | 0.219 | -0.69 | 0.766 | -0.659 | 0.359 | 0.861 |
| Impala | During - Pre == 0 | -0.161 | 0.056 | -2.873 | 0.011 * | -0.291 | -0.030 | **0.851** |
| | Post - Pre == 0 | -0.153 | 0.059 | -2.605 | 0.024 * | -0.291 | -0.016 | **0.858** |
| | Post - During == 0 | 0.007 | 0.037 | 0.190 | 0.980 | -0.080 | 0.094 | 1.007 |
| All Parasites | During - Pre == 0 | 2.121 | 0.685 | 3.099 | 0.00527 ** | 0.528 | 3.714 | **8.339** |
| | Post - Pre == 0 | 0.9898 | 0.722 | 1.372 | 0.348 | -0.689 | 2.669 | 2.691 |
| | Post - During == 0 | -1.131 | 0.456 | -2.480 | 0.034 * | -2.193 | -0.0694 | **0.323** |

## Table S13. Observational System Log-Ratio LMM Results

Coefficients and their 95% confidence intervals for best models of the log ratio of dung and parasite density at water sources versus matrix sites. Coefficients indicating increased dung and parasite density at water relative to matrix sites are shaded green, while those indicating decreased relative density are shaded orange. The intercept corresponds to the log ratio in dung density when all covariates are zero. Significant two-sided p-values (Fisher t-tests) are shown in bold.

| Species | Intercept | Distance | MAP | Rain | Distance:MAP | Rain:MAP | σ/Site/Period |
|---|---|---|---|---|---|---|---|
| All | 6.86<br>5.40<br>4.36, 9.36<br>4.49, 9.03<br>**<0.001** | -3.65<br>-2.56<br>-6.45, -0.85<br>-6.15, -1.05<br>**0.01** | -0.88<br>-3.90<br>-1.32, -0.44<br>-1.27, -0.46<br>**<0.001** | -0.92<br>-4.55<br>-1.33, -0.52<br>-1.32, -0.51<br>**<0.001** | 0.51<br>2.01<br>0.011, 1.00<br>0.049, 0.94<br>**0.045** | | 7.36<br>0.01<br>0.00 |
| Cow | 2.42<br>4.35<br>1.31, 3.59<br>1.74, 3.34<br>**<0.001** | -0.63<br>-6.01<br>-0.83, -0.42<br>-0.83, -0.43<br>**<0.001** | -0.23<br>-2.40<br>-0.43, -0.04<br>-0.39, -0.11<br>**0.02** | -0.33<br>-2.17<br>-0.66, -0.033<br>-0.67, -0.068<br>**0.03** | | | 3.28<br>0.03<br>0.02 |
| Elephant | 3.91<br>4.92<br>2.27, 5.51<br>2.82, 4.85<br>**<0.001** | -0.718<br>-4.90<br>-1.01, -0.43<br>-1.01, -0.43<br>**<0.001** | -0.477<br>-3.46<br>-0.75, -0.19<br>-0.627, -0.300<br>**0.002** | | | | 6.42<br>0.07<br>0.01 |
| Zebra | 2.16<br>2.66<br>0.56, 3.82<br>0.85, 3.86<br>**0.009** | | -0.36<br>-2.49<br>-0.65, -0.075<br>-0.67, -0.13<br>**0.014** | -3.01<br>-2.66<br>-5.25, -0.78<br>-5.13, -1.36<br>**0.008** | | 0.48<br>2.44<br>0.092, 0.88<br>0.20, 0.87<br>**0.015** | 2.59<br>0.03<br>0.00 |
| Impala | -0.56<br>-2.45<br>-1.01, -0.11<br>-1.06, -0.08<br>**0.015** | 0.83<br>3.44<br>0.36, 1.31<br>0.28, 1.44<br>**<0.001** | 0.11<br>2.62<br>0.026, 0.19<br>0.024, 0.19<br>**0.009** | | -0.15<br>-3.48<br>-0.23, -0.065<br>-0.25, -0.056<br>**<0.001** | | 0.21<br>0.00<br>0.00 |
| Buffalo | 0.28<br>3.21<br>0.092, 0.48<br>0.15, 0.45<br>**0.008** | | | -0.30<br>-2.88<br>-0.50, -0.095<br>-0.55, -0.093<br>**0.004** | | | 1.38<br>0.00<br>0.02 |
| Giraffe | 0.14<br>2.84<br>0.04, 0.24<br>0.063, 0.22<br>**0.01** | | | -0.18<br>-2.82<br>-0.32, -0.054<br>-0.32, -0.055<br>**0.007** | | | 0.61<br>0.01<br>0.00 |
| All Parasites | 9.42<br>5.94<br>6.31, 12.53<br>6.69, 11.79<br>**<0.001** | -1.48<br>-4.33<br>**-2.15, -0.81**<br>**-2.13, -0.83**<br>**<0.001** | -0.99<br>-3.58<br>-1.54, -0.45<br>-1.45, -0.52<br>**0.001** | -2.11<br>-4.74<br>-2.98, -1.24<br>-2.92, -1.06<br>**<0.001** | | | 35.07<br>0.11<br>0.00 |

**Legend:**

| |
|---|
| Estimate<br>t-value<br>95% Profile interval<br>95% Bias-corrected bootstrap interval<br>Pr(>\|t\|) |

## Table S14. Experimental System GLMMs for Matrix Sites

GLMM hurdle model coefficients for comparisons between matrix sites and filled water pans throughout the experimental study at Ol Pejeta Conservancy. Coefficients that indicate increased and decreased dung at water sources relative to matrix sites are shaded in blue and orange respectively. There were no significant interactions between filled pans and experimental status for any species except impala. Significant two-sided p-values (Fisher t-tests) are shown in bold. Marginal (M) and conditional (C) $R^2$ values are provided.

| | All | Elephant | Cow | Zebra | Buffalo | Impala | Giraffe | Parasites |
|---|---|---|---|---|---|---|---|---|
| **Conditional Model** | | | | | | | | |
| Intercept (Pre, Dry, 0m) | 3.86<br>2.08, 5.65<br>**<0.001** | 4.22<br>1.80, 6.65<br>**0.001** | 3.16<br>1.58, 4.75<br>**<0.001** | 3.8<br>2.59, 5.01<br>**<0.001** | 4.28<br>2.84, 5.73<br>**<0.001** | 1.05<br>0.20, 1.90<br>**0.016** | 1.44<br>0.92, 1.96<br>**<0.001** | 24.95<br>13.3, 36.56<br>**<0.001** |
| During | -0.94<br>-2.8, 0.92<br>0.323 | -0.42<br>-2.46, 1.6<br>0.686 | -0.59<br>-2.1, 0.96<br>0.457 | -0.79<br>-1.98, 0.4<br>0.197 | -0.77<br>-2.16, 0.6<br>0.278 | -0.51<br>-1.44, 0.42<br>0.281 | -0.06<br>-0.44, 0.3<br>0.742 | -5.42<br>-17.45, 6.6<br>0.377 |
| Post | -0.92<br>-2.9, 1.05<br>0.36 | -0.32<br>-2.4, 1.77<br>0.767 | -1.15<br>-2.8, 0.45<br>0.16 | -0.63<br>-1.9, 0.67<br>0.345 | -1.36<br>-2.85, 0.1<br>0.074 | -0.62<br>-1.60, 0.35<br>0.211 | -0.03<br>-0.4, 0.36<br>0.884 | -4.76<br>-17.45, 7.9<br>0.462 |
| Filled | 2.35<br>1.47, 3.24<br>**<0.001** | 1.17<br>-1.0, 3.37<br>0.298 | 2.29<br>1.02, 3.56<br>**<0.001** | 0.23<br>-1.2, 1.66<br>0.755 | 0.32<br>-0.97, 1.6<br>0.622 | 0.25<br>-0.01, 0.50<br>0.062 | -0.61<br>-1.84, 0.6<br>0.331 | 14.15<br>7.66, 20.64<br>**<0.001** |
| ln(Dist+1) | -0.01<br>-0.15, 0.1<br>0.847 | 0.18<br>-0.2, 0.56<br>0.333 | 0.03<br>-0.14, 0.2<br>0.752 | -0.12<br>-0.3, 0.04<br>0.144 | 0.02<br>-0.16, 0.2<br>0.814 | -0.02<br>-0.04, 0.01<br>0.133 | 0.02<br>-0.07, 0.1<br>0.662 | -0.44<br>-1.40, 0.52<br>0.37 |
| During: Filled | 0.1<br>-0.6, 0.77<br>0.773 | -0.12<br>-1.7, 1.45<br>0.877 | -0.53<br>-1.65, 0.6<br>0.351 | -0.04<br>-0.78, 0.7<br>0.919 | 0.43<br>-0.47, 1.3<br>0.353 | -0.38<br>-0.61, -0.15<br>**0.001** | 0.25<br>-0.38, 0.9<br>0.436 | -0.24<br>-5.12, 4.64<br>0.924 |
| Post: Filled | 0.12<br>-0.6, 0.83<br>0.739 | -1.3<br>-2.9, 0.26<br>0.102 | -0.1<br>-1.22, 1.0<br>0.867 | -0.54<br>-1.39, 0.3<br>0.215 | 0.85<br>-0.3, 1.95<br>0.133 | -0.29<br>-0.52, -0.06<br>**0.012** | 0.21<br>-0.4, 0.86<br>0.524 | -1.37<br>-6.52, 3.78<br>0.602 |
| Filled: ln(Dist+1) | -0.41<br>-0.58, -0.2<br>**<0.001** | -0.18<br>-0.6, 0.24<br>0.404 | -0.28<br>-0.47, -0.1<br>**0.005** | 0.01<br>-0.3, 0.33<br>0.937 | -0.22<br>-0.5, 0.06<br>0.122 | 0.03<br>-0.00, 0.06<br>0.096 | 0.07<br>-0.17, 0.3<br>0.569 | -1.5<br>-2.74, -0.25<br>**0.018** |
| **Zero-Inflated Model** | | | | | | | | |
| (Int.) | -1.18<br>-2.7, 0.35<br>0.131 | 2.04<br>0.61, 3.46<br>**0.005** | 3.16<br>1.61, 4.72<br>**<0.001** | 0.15<br>-1.3, 1.57<br>0.838 | 1.39<br>-0.23, 3.0<br>0.092 | 1.5<br>-15.2, 18.2<br>0.86 | 1.37<br>0.01, 2.72<br>**0.048** | -1.14<br>-2.55, 0.26<br>0.11 |
| During | 0.82<br>-0.7, 2.37<br>0.297 | 0.52<br>-0.75, 1.8<br>0.422 | -1<br>-2.4, 0.42<br>0.167 | 1.06<br>-0.3, 2.44<br>0.131 | 1.11<br>-0.37, 2.6<br>0.141 | -10.09<br>-28.4, 8.23<br>0.28 | -0.08<br>-1.37, 1.2<br>0.905 | 0.93<br>-0.49, 2.36<br>0.199 |
| Post | 0.5<br>-1.1, 2.14<br>0.547 | -0.27<br>-1.6, 1.04<br>0.686 | -1.58<br>-3.06, -0.1<br>**0.035** | 1.51<br>0.03, 2.98<br>**0.045** | 1.05<br>-0.5, 2.63<br>0.19 | -11.16<br>-29.63, 7.3<br>0.236 | -0.1<br>-1.5, 1.26<br>0.881 | 0.61<br>-0.90, 2.11<br>0.43 |
| Filled | -1.9<br>-3.3, -0.48<br>**0.009** | -2.31<br>-3.6 – -1.1<br>**<0.001** | -4.52<br>-5.9 – -3.1<br>**<0.001** | 2.84<br>1.6 – 4.09<br>**<0.001** | -0.36<br>-1.7 – 1.0<br>0.596 | 1.35<br>-0.42 – 3.1<br>0.135 | 2.07<br>0.45, 3.69<br>**0.012** | -2.02<br>-3.36, -0.69<br>**0.003** |

| | | | | | | | | |
|---|---|---|---|---|---|---|---|---|
| ln(Dist+1) | -0.05<br>-0.2, 0.09<br>0.51 | -0.03<br>-0.2, 0.16<br>0.734 | -0.14<br>-0.3, 0.04<br>0.121 | 0.09<br>-0.06, 0.2<br>0.223 | -0.03<br>-0.2, 0.16<br>0.721 | 0.02<br>-0.23, 0.27<br>0.889 | 0.05<br>-0.1, 0.2<br>0.522 | -0.04<br>-0.16, 0.09<br>0.589 |
| During: Filled | -0.5<br>-1.5, 0.49<br>0.325 | -0.15<br>-1.1, 0.83<br>0.766 | 0.18<br>-0.86, 1.2<br>0.737 | -0.75<br>-1.5, 0.01<br>0.052 | -0.03<br>-0.98, 0.9<br>0.944 | -0.79<br>-1.82, 0.23<br>0.128 | 0.11<br>-0.9, 1.12<br>0.827 | -0.45<br>-1.38, 0.48<br>0.342 |
| Post: Filled | -0.78<br>-1.87, 0.3<br>0.163 | -0.44<br>-1.4, 0.54<br>0.378 | -0.14<br>-1.2, 0.94<br>0.805 | -0.51<br>-1.35, 0.3<br>0.231 | 0.86<br>-0.29, 2.0<br>0.141 | 0.24<br>-0.89, 1.37<br>0.676 | 0.02<br>-1.0, 1.06<br>0.976 | -0.71<br>-1.71, 0.30<br>0.168 |
| Filled: ln(Dist+1) | 0.3<br>0.03, 0.57<br>**0.028** | 0.49<br>0.26, 0.73<br>**<0.001** | 0.67<br>0.43, 0.92<br>**<0.001** | -0.81<br>-1.1, -0.55<br>**<0.001** | 0.22<br>-0.06, 0.5<br>0.12 | -0.37<br>-0.76, 0.02<br>0.06 | -0.43<br>-0.74, -0.1<br>**0.007** | 0.33<br>0.08, 0.58<br>**0.01** |
| **Random Effects** | | | | | | | | |
| $\sigma^2$ | 2.9 | 3.16 | 1.37 | 1.6 | 1.01 | 0.09 | 0.36 | 161.88 |
| $\tau_{00}$ Site | 0.06 | 0 | 0.06 | 0.01 | 0.02 | 0 | 0 | 1.71 |
| $\tau_{00}$ Period | 0.7 | 0.56 | 0.3 | 0.24 | 0.35 | 0.18 | 0 | 28.58 |
| N | 1440 | 1440 | 1440 | 1440 | 1440 | 1440 | 1440 | 1440 |
| M. $R^2$ / C. $R^2$ | 0.097 / 0.285 | | 0.178 / 0.347 | 0.055 / 0.182 | 0.085 / 0.329 | 0.174 / 0.724 | | 0.098 / 0.240 |

**Table S15. Experimental System Estimated Exposures**

Estimates of parasite exposure ratios at permanently filled water pans relative to matrix sites for each focal herbivore species at Ol Pejeta Conservancy. P-value adjustment for two-sided *t*-tests was performed using the Holm correction for multiple comparisons. Significant ($p_{adjusted} < 0.05$; in bold) and marginally significant ($p_{adjusted} < 0.1$) ratios are shown in red.

| Species | Scenario | Exposure ratio | SE | df | *t* ratio | Unadjusted *p* | Adjusted *p* |
|---|---|---|---|---|---|---|---|
| Buffalo | Low | **8.00** | 5.45 | 45 | 3.05 | 0.004 | 0.008 |
| | Equal | 4.00 | 2.72 | 45 | 2.04 | 0.047 | 0.095 |
| | High | 2.00 | 1.36 | 45 | 1.02 | 0.313 | 0.625 |
| | Very High | 0.40 | 0.27 | 45 | -1.35 | 0.185 | 0.651 |
| Cattle | Low | **285.19** | 158.67 | 45 | 10.16 | <0.001 | <0.001 |
| | Equal | **142.59** | 78.39 | 45 | 9.02 | <0.001 | <0.001 |
| | High | **71.30** | 38.84 | 45 | 7.83 | <0.001 | <0.001 |
| | Very High | **14.26** | 7.72 | 45 | 4.91 | <0.001 | <0.001 |
| Elephant | Low | **134.76** | 79.81 | 45 | 8.28 | <0.001 | <0.001 |
| | Equal | **67.38** | 39.53 | 45 | 7.18 | <0.001 | <0.001 |
| | High | **33.69** | 19.62 | 45 | 6.04 | <0.001 | <0.001 |
| | Very High | **6.74** | 3.91 | 45 | 3.29 | 0.002 | 0.01 |
| Giraffe | Low | 5.44 | 4.91 | 45 | 1.88 | 0.067 | 0.067 |
| | Equal | 2.72 | 2.47 | 45 | 1.10 | 0.277 | 0.277 |
| | High | 1.36 | 1.24 | 45 | 0.34 | 0.738 | 0.738 |
| | Very High | 0.27 | 0.25 | 45 | -1.42 | 0.163 | 0.651 |
| Impala | Low | **16.19** | 11.61 | 45 | 3.88 | <0.001 | 0.001 |
| | Equal | **8.09** | 5.80 | 45 | 2.92 | 0.005 | 0.016 |
| | High | 4.05 | 2.89 | 45 | 1.95 | 0.057 | 0.170 |
| | Very High | 0.81 | 0.58 | 45 | -0.30 | 0.769 | 0.769 |
| Zebra | Low | **39.48** | 21.76 | 45 | 6.67 | <0.001 | <0.001 |
| | Equal | **19.74** | 10.76 | 45 | 5.47 | <0.001 | <0.001 |
| | High | **9.87** | 5.34 | 45 | 4.23 | <0.001 | <0.001 |
| | Very High | 1.97 | 1.06 | 45 | 1.26 | 0.214 | 0.651 |

**Table S16. Observational System Estimated Exposures**

Estimates of parasite exposure ratios at dams relative to matrix sites for each focal herbivore species at Mpala Research Centre. P-value adjustment for two-sided *t*-tests was performed using the Holm correction for multiple comparisons. Significant ($p_{adjusted} < 0.05$; in bold) and marginally significant ($p_{adjusted} < 0.1$) ratios are shown in red.

| Species | Scenario | Exposure ratio | SE | df | *t* ratio | Unadjusted *p* | Adjusted *p* |
|---|---|---|---|---|---|---|---|
| Buffalo | Low | **168.81** | 315.11 | 129 | 2.75 | 0.007 | 0.027 |
| | Equal | 85.03 | 156.61 | 129 | 2.41 | 0.017 | 0.086 |
| | High | 42.84 | 78.02 | 129 | 2.06 | 0.041 | 0.205 |
| | Very High | 8.79 | 15.71 | 129 | 1.22 | 0.226 | 0.783 |
| Cattle | Low | **25.32** | 28.74 | 129 | 2.85 | 0.005 | 0.026 |
| | Equal | 12.87 | 14.39 | 129 | 2.28 | 0.024 | 0.096 |
| | High | 6.58 | 7.27 | 129 | 1.70 | 0.091 | 0.364 |
| | Very High | 1.41 | 1.55 | 129 | 0.31 | 0.754 | 1.00 |
| Elephant | Low | **76.74** | 78.29 | 129 | 4.25 | <0.001 | <0.001 |
| | Equal | **38.59** | 38.59 | 129 | 3.65 | <0.001 | 0.002 |
| | High | **19.43** | 19.12 | 129 | 3.02 | 0.003 | 0.019 |
| | Very High | 3.96 | 3.83 | 129 | 1.42 | 0.157 | 0.783 |
| Giraffe | Low | 6.89 | 17.42 | 129 | 0.76 | 0.446 | 0.893 |
| | Equal | 3.42 | 8.61 | 129 | 0.49 | 0.627 | 1.00 |
| | High | 1.70 | 4.28 | 129 | 0.21 | 0.833 | 1.00 |
| | Very High | 0.34 | 0.86 | 129 | -0.43 | 0.67 | 1.00 |
| Impala | Low | 1.10 | 1.74 | 129 | 0.06 | 0.952 | 0.952 |
| | Equal | 0.59 | 0.93 | 129 | -0.33 | 0.739 | 1.00 |
| | High | 0.32 | 0.50 | 129 | -0.73 | 0.469 | 1.00 |
| | Very High | 0.08 | 0.12 | 129 | -1.61 | 0.109 | 0.654 |
| Zebra | Low | 3.44 | 3.78 | 129 | 1.12 | 0.264 | 0.793 |
| | Equal | 1.83 | 2.01 | 129 | 0.55 | 0.581 | 1.00 |
| | High | 0.98 | 1.07 | 129 | -0.02 | 0.986 | 1.00 |
| | Very High | 0.23 | 0.26 | 129 | -1.32 | 0.189 | 0.783 |

**Table S17: Landscape-Level Exposure Comparisons**

Estimated percentage of parasite exposures occurring near water for each species after accounting for the area and distribution of water sources across the landscape. Raw ratio reflects values reported in Tables S15 and S16; Landscape Ratio shows the proportion of the total landscape located within 150m of non-riparian water, and % Water shows the percentage of all estimated exposures that occur near water for each species. Equal mortality values are shown in bold to highlight results under an assumption of no change in parasite mortality near water versus the surrounding landscape.

| | Parasite Mortality Scenario | **Observational System Exposures** | | | **Experimental System Exposures** | | |
|---|---|---|---|---|---|---|---|
| | | Raw Ratio | Landscape Ratio | % Water | Raw Ratio | Landscape Ratio | % Water |
| Elephant | Low | 77 | 1.20 | 54.64 | 135 | 3.62 | 78.35 |
| | **Equal** | **39** | **0.61** | **37.89** | **67** | **1.80** | **64.23** |
| | High | 19 | 0.30 | 22.91 | 34 | 0.91 | 47.68 |
| | Very High | 4 | 0.06 | 5.89 | 7 | 0.19 | 15.80 |
| Cattle | Low | 25 | 0.39 | 28.11 | 285 | 7.64 | 88.42 |
| | **Equal** | **13** | **0.20** | **16.90** | **143** | **3.83** | **79.31** |
| | High | 7 | 0.11 | 9.87 | 71 | 1.90 | 65.55 |
| | Very High | 1 | 0.02 | 1.54 | 14 | 0.38 | 27.28 |
| Zebra | Low | 3 | 0.05 | 4.48 | 39 | 1.05 | 51.10 |
| | **Equal** | **2** | **0.03** | **3.03** | **20** | **0.54** | **34.90** |
| | High | 1 | 0.02 | 1.54 | 10 | 0.27 | 21.14 |
| | Very High | 0.2 | 0.00 | 0.31 | 2 | 0.05 | 5.09 |
| Impala | Low | 1 | 0.02 | 1.54 | 16 | 0.43 | 30.01 |
| | **Equal** | **0.6** | **0.01** | **0.93** | **8** | **0.21** | **17.65** |
| | High | 0.3 | 0.00 | 0.47 | 4 | 0.11 | 9.68 |
| | Very High | 0.08 | 0.00 | 0.12 | 0.8 | 0.02 | 2.10 |
| Buffalo | Low | 169 | 2.64 | 72.55 | 8 | 0.21 | 17.65 |
| | **Equal** | **85** | **1.33** | **57.07** | **4** | **0.11** | **9.68** |
| | High | 43 | 0.67 | 40.21 | 2 | 0.05 | 5.09 |
| | Very High | 9 | 0.14 | 12.34 | 0.4 | 0.01 | 1.06 |
| Giraffe | Low | 7 | 0.11 | 9.87 | 5 | 0.13 | 11.82 |
| | **Equal** | **3** | **0.05** | **4.48** | **3** | **0.08** | **7.44** |
| | High | 2 | 0.03 | 3.03 | 1 | 0.03 | 2.61 |
| | Very High | 0.3 | 0.00 | 0.47 | 0.3 | 0.01 | 0.80 |

## SI References


1. C. L. Nunn, S. M. Altizer, The global mammal parasite database: An online resource for infectious disease records in wild primates. *Evol. Anthropol. Issues, News, Rev.* **14**, 1–2 (2005).
2. D. I. Gibson, R. A. Bray, E. A. Harris, Host-Parasite Database of the Natural History Museum, London (2005).
3. M. C. Round, *Check list of the helminth parasites of African mammals of the orders Carnivora, Tubulidentata, Proboscidea, Hyra-coidea, Artiodactyla and Perissodactyla.* (Farnham Royal: Commonwealth Agricultural Bureaux, 1968).
4. M. T. Fox, Gastrointestinal Parasites of Sheep and Goats - Digestive System. *Merck Vet. Man.* (2014).
5. M. T. Fox, Gastrointestinal Parasites of Cattle - Digestive System. *Merck Vet. Man.* (2014).
6. M. Thorn, D. M. Scott, M. Green, P. W. Bateman, E. Z. Cameron, Estimating Brown Hyaena Occupancy Using Baited Camera Traps. *South African J. Wildl. Res.* **39**, 1–10 (2009).
7. M. Chame, Terrestrial Mammal Feces: A Morphometric Summary and Description. *Mem. Inst. Oswaldo Cruz* **98**, 71–94 (2003).
8. A. Kuznetsova, P. B. Brockhoff, R. H. B. Christensen, lmerTest Package: Tests in Linear Mixed Effects Models. *J. Stat. Softw.* **82** (2017).
9. H.-T. Thai, F. Mentré, N. H. G. Holford, C. Veyrat-Follet, E. Comets, A comparison of bootstrap approaches for estimating uncertainty of parameters in linear mixed-effects models. *Pharm. Stat.* **12**, 129–140 (2013).
10. M. L. Delano, S. A. Mischler, W. J. Underwood, "Biology and Diseases of Ruminants: Sheep, Goats, and Cattle" in *Laboratory Animal Medicine*, (Elsevier, 2002), pp. 519–614.
11. E. Ghadirian, F. Arfaa, First report of human infection with Haemonchus contortus, Ostertagia ostertagi, and Marshallagia marshalli (family trichostrongylidae) in Iran. *J. Parasitol.* **59**, 1144–1145 (1973).
12. R. K. Keith, The pathogenicity of experimental infections of cooperia pectinata ransom, 1907 in calves. *Aust. J. Agric. Res.* **18**, 861–864 (1967).
13. J. E. Alicata, F. T. Lynd, Growth rate and other signs of infection in calves experimentally infected with Cooperia punctata. *Am. J. Vet. Res.* **22**, 704–707 (1961).
14. R. L. Coop, A. R. Sykes, K. W. Angus, The pathogenicity of daily intakes of Cooperia oncophora larvae in growing calves. *Vet. Parasitol.* **5**, 261–269 (1979).
15. T. E. Gibson, Studies on Trichostrongylus axei IV Factors in the causation of pathogenic effects by T. axei. *J. Comp. Path.* **65**, 317–324 (1955).
16. G. Cancrini, G. Boemi, A. Iori, A. Corselli, Infestazioni umane da Trichostrongylus axei, T. capricola e T. vitrinus: prima segnalazione in Italia. *Parassitologia* **24**, 145–149 (1982).
17. I. Ziomko, Experimental invasion of Strongyloides papillosus (Wedl, 1856) in sheep. *Bull. Vet. Inst. Pulawy* **44**, 179–186 (2000).
18. P. H. Holmes, Pathogenesis of trichostrongylosis. *Vet. Parasitol.* **18**, 89–101 (1985).
19. M. Sato, *et al.*, Human trichostrongylus colubriformis infection in a rural village in Laos. *Am. J. Trop. Med. Hyg.* **84**, 52–54 (2011).
20. F. Roeber, A. R. Jex, R. B. Gasser, "Next-Generation Molecular-Diagnostic Tools for Gastrointestinal Nematodes of Livestock, with an Emphasis on Small Ruminants. A Turning Point?" in *Advances in Parasitology*, (Academic Press, 2013), pp. 267–333.
21. S. Dalal, A. Prasad, A. Nasir, V. K. Saini, Cross antigenicity of immunodominant polypeptides of somatic antigen of Oesophagostomum columbianum with other helminths by western blotting. *Vet. World* **8**, 1279–1285 (2015).
22. J. Owen, D. Slocombe, Pathogenesis of helminths in equines. *Vet. Parasitol.* **18**, 139–153 (1985).
23. X. Cao, A. N. Vidyashankar, M. K. Nielsen, Association between large strongyle genera in larval cultures--using rare-event poisson regression. *Parasitology* **140**, 1246–1251 (2013).
24. Z. Ansari, K. Suresh Singh, Effect of gamma-irradiation on the survival and development of the infective larvae of the hookworm, Gaigeria pachyscelis*. *J. ofHelminthology* **52**, 283–286 (2020).
25. H. Herlich, L. C. Gasbarre, F. W. Douvres, Infectivity and pathogenicity of three isolates of Ostertagia ostertagi in cattle. *Vet. Parasitol.* **16**, 253–260 (1984).
26. F. C. Fávero, *et al.*, Experimental infection of calves with Haemonchus placei or Haemonchus

contortus: Assessment of clinical, hematological and biochemical parameters and histopathological characteristics of abomasums. *Exp. Parasitol.* **170**, 125–134 (2016).
27. I. J. East, C. J. Fitzgerald, D. A. Berrie, Oesophagostomum radiatum: the effect of different adjuvants on vaccination with a partially purified protective antigen. *Vet. Parasitol.* **49**, 191–200 (1993).
28. C. R. Wang, J. F. Gao, X. Q. Zhu, Q. Zhao, Characterization of Bunostomum trigonocephalum and Bunostomum phlebotomum from sheep and cattle by internal transcribed spacers of nuclear ribosomal DNA. *Res. Vet. Sci.* **92**, 99–102 (2012).
29. A. Samizadeh-Yazd, A. C. Todd, Observations on the pathogenic effects of Nematodirus helvetianus in dairy calves. *Am. J. Vet. Res.* **40**, 48–51 (1979).
30. D. Van Aken, *et al.*, Pathophysiological aspects of Mecistocirrus digitatus (Nematoda: Trichostrongylidae) infection in calves. *Vet. Parasitol.* **69**, 255–263 (1997).
31. M. J. Stear, S. C. Bishop, N. G. Henderson, I. Scott, A key mechanism of pathogenesis in sheep infected with the nematode Teladorsagia circumcincta. *Anim. Heal. Res. Rev.* **4** (2003).
32. K. Ashrafi, M. Sharifdini, Z. Heidari, B. Rahmati, E. B. Kia, Zoonotic transmission of Teladorsagia circumcincta and Trichostrongylus species in Guilan province, northern Iran: Molecular and morphological characterizations. *BMC Infect. Dis.* **20**, 1–9 (2020).
33. R. P. Herd, The pathogenic importance of Chabertia ovina (Fabricius, 1788) in experimentally infected sheep. *Int. J. Parasitol.* **1**, 251–263 (1971).
34. L. Seghetti, C. M. Senger, Experimental infections in lambs with Nematodirus spathiger. *Am. J. Vet. Res.* **19**, 642–644 (1958).
35. R. L. Coop, K. W. Angus, A. R. Sykes, Chronic infection with Trichostrongylus vitrinus in sheep. Pathological changes in the small intestine. *Res. Vet. Sci.* **26**, 363–371 (1979).
36. B. R. Beechler, H. Broughton, A. Bell, V. O. Ezenwa, A. E. Jolles, Innate immunity in free-ranging African buffalo (Syncerus caffer): Associations with parasite infection and white blood cell counts. *Physiol. Biochem. Zool.* **85**, 255–264 (2012).
37. A. Caron, P. C. Cross, J. T. du Toit, Ecological implications of bovine tuberculosis in African buffalo herds. *Ecol. Appl.* **13**, 1338–1345 (2003).
38. V. O. Ezenwa, Habitat overlap and gastrointestinal parasitism in sympatric African bovids. *Parasitology* **126**, 379–388 (2003).
39. V. O. Ezenwa, Interactions among host diet, nutritional status and gastrointestinal parasite infection in wild bovids. *Int. J. Parasitol.* **34**, 535–542 (2004).
40. V. O. Ezenwa, A. E. Jolles, Horns honestly advertise parasite infection in male and female African buffalo. *Anim. Behav.* **75**, 2013–2021 (2008).
41. E. E. Gorsich, V. O. Ezenwa, A. E. Jolles, Nematode-coccidia parasite co-infections in African buffalo: Epidemiology and associations with host condition and pregnancy. *Int. J. Parasitol. Parasites Wildl.* **3**, 124–34 (2014).
42. B. L. Penzhorn, Coccidian oocyst and nematode egg counts of free-ranging African buffalo (Syncerus caffer) in the Kruger National Park, South Africa. *J. S. Afr. Vet. Assoc.* **71**, 106–108 (2000).
43. A. D. Agyei, Epidemiological observations on helminth infections of calves in southern Ghana. *Trop. Anim. Health Prod.* **23**, 134–140 (1991).
44. D. L. Brito, *et al.*, Effect of alternate and simultaneous grazing on endoparasite infection in sheep and cattle. *Rev. Bras. Parasitol. Veterinária* **22**, 485–494 (2013).
45. H. Degefu, C. Abera, M. Yohannes, T. Tolosa, Gastrointestinal helminth infections in small-scale dairy cattle farms of Jimma town, Ethiopia. *Ethiop. J. Appl. Sci. Technol.* **2**, 31–37 (2011).
46. W. Kabaka, G. Gitau, P. Kitala, N. Maingi, J. Van Leeuwen, The prevalence of gastrointestinal nematode infection and their impact on cattle in Nakuru and Mukurweini districts of Kenya. *Ethiop. Vet. J.* **17**, 95 (2014).
47. P. W. N. Kanyari, J. M. Kagira, J. R. L. Mhoma, Prevalence of endoparasites in cattle within urban and peri-urban areas of Lake Victoria Basin, Kenya with special reference to zoonotic potential. *Sci. Parasitol.* **11**, 171–178 (2010).
48. J. D. Keyyu, N. C. Kyvsgaard, J. Monrad, A. A. Kassuku, Epidemiology of gastrointestinal nematodes in cattle on traditional, small-scale dairy and large-scale dairy farms in Iringa district, Tanzania. *Vet. Parasitol.* **127**, 285–294 (2005).
49. S. E. Knafo, "Distribution of gastrointestinal parasites of Grevy's zebras, plains zebras, domestic

donkeys, and domestic cattle in the Samburu landscape, Kenya," Tufts University. (2008).
50. T. Ndlovu, M. Chimonyo, V. Muchenje, Monthly changes in body condition scores and internal parasite prevalence in Nguni, Bonsmara and Angus steers raised on sweetveld. *Trop. Anim. Health Prod.* **41**, 1169–1177 (2009).
51. P. Sun, T. Wronski, J. D. Bariyanga, A. Apio, Gastro-intestinal parasite infections of Ankole cattle in an unhealthy landscape: An assessment of ecological predictors. *Vet. Parasitol.* **252**, 107–116 (2018).
52. R. M. Waruiru, *et al.*, The Prevalence and Intensity of Helminth and Coccidial Infections in Dairy Cattle in Central Kenya. *Vet. Res. Commun.* **24**, 39–53 (2000).
53. L. Baines, E. R. Morgan, M. Ofthile, K. Evans, Occurrence and seasonality of internal parasite infection in elephants, Loxodonta africana, in the Okavango Delta, Botswana. *Int. J. Parasitol. Parasites Wildl.* **4**, 43–48 (2015).
54. K. N. Brumfitt, "Assesment of body condition of African elephants (Loxodonta africana) in North East of Etosha National Park, Namibia: How it relates to strongyle parasite eggs counts and nutrition value of feed," University of Namibia. (2005).
55. E. King'ori, *et al.*, Patterns of helminth infection in Kenyan elephant populations. *Parasites and Vectors* **13**, 1–12 (2020).
56. J. M. Parker, S. Z. Goldenberg, D. Letitiya, G. Wittemyer, Strongylid infection varies with age, sex, movement and social factors in wild African elephants. *Parasitology* **147**, 348–359 (2020).
57. M. I. Thurber, *et al.*, Effects of rainfall, host demography, and musth on strongyle fecal egg counts in African elephants (Loxodonta Africana) in Namibia. *J. Wildl. Dis.* **47**, 172–181 (2011).
58. C. P. Melbourne, Observations on the Treatment of Endoparasites of Giraffes at Longleat Safari Park. *J. Zoo Anim. Med.* **9**, 146 (1978).
59. K. VanderWaal, G. P. Omondi, V. Obanda, Mixed-host aggregations and helminth parasite sharing in an East African wildlife-livestock system. *Vet. Parasitol.* **205**, 224–232 (2014).
60. K. S. Nalubamba, N. B. Mudenda, M. R. Malamo, A seasonal survey of gastrointestinal parasites in captive wild impala antelope on a game facility south of Lusaka, Zambia. *J. Helminthol.* **86**, 418–425 (2012).
61. M. Ocaido, L. Siefert, J. Baranga, Seasonal changes of impala (Aepyceros melampus, Lichtenstein, 1812) faecal helminth egg counts through a one-year period. *Afr. J. Ecol.* **37**, 327–333 (1999).
62. M. S. Mwatenga, "Gastrointestinal parasites infesting Grevy's zebra (Equus Grevyi) in the Samburu landscape in Samburu county," Kenyatta University. (2017).
63. D. I. Rubenstein, *Ecology, social behavior, and conservation in zebras* (Academic Press, 2010).
64. M. C. Fugazzola, L. Stancampiano, Host social rank and parasites: Plains zebra (Equus quagga) and intestinal helminths in Uganda. *Vet. Parasitol.* **188**, 115–119 (2012).
65. R. C. Krecek, R. K. Reinecke, F. S. Malan, Studies on the parasites of zebras. V. Nematodes of the Burchell's and Hartmann's mountain zebras from the Etosha National Park, South West Africa/Namibia. *Onderstepoort J. Vet. Res.* **54**, 71–78 (1987).
66. W. C. Turner, "The Ecology of Orally Ingested Parasites in Ungulates of Etosha National Park," University of California, Berkeley. (2009).
67. E. N. Wambwa, W. O. Ogara, D. Mudakha, A comparative study of gastrointestinal parasites between ranched and free ranging Burchell's zebra (Equus burchelli antiquorum) in Isiolo district, Kenya. *J. Vet. Sci.* **5**, 215–220 (2004).
