# Supplementary material for: Water sources aggregate parasites with increasing effects in more arid conditions
Source: Nat Commun. 2021 Dec 3;12:7066. doi: 10.1038/s41467-021-27352-y (PMC8642388; doi:10.1038/s41467-021-27352-y)
Supplement: Supplementary file 3 — Reporting Summary [file 41467_2021_27352_MOESM3_ESM.pdf]

nature research

Corresponding author(s): Georgia Titcomb

Last updated by author(s): Oct 27, 2021

Reporting Summary

Nature Research wishes to improve the reproducibility of the work that we publish. This form provides structure for consistency and transparency in reporting. For further information on Nature Research policies, see our Editorial Policies and the Editorial Policy Checklist.

Statistics

For all statistical analyses, confirm that the following items are present in the figure legend, table legend, main text, or Methods section.

n/a	Confirmed	
☐	☒	The exact sample size (*n*) for each experimental group/condition, given as a discrete number and unit of measurement
☐	☒	A statement on whether measurements were taken from distinct samples or whether the same sample was measured repeatedly
☐	☒	The statistical test(s) used AND whether they are one- or two-sided<br>*Only common tests should be described solely by name; describe more complex techniques in the Methods section.*
☐	☒	A description of all covariates tested
☐	☒	A description of any assumptions or corrections, such as tests of normality and adjustment for multiple comparisons
☐	☒	A full description of the statistical parameters including central tendency (e.g. means) or other basic estimates (e.g. regression coefficient) AND variation (e.g. standard deviation) or associated estimates of uncertainty (e.g. confidence intervals)
☐	☒	For null hypothesis testing, the test statistic (e.g. *F*, *t*, *r*) with confidence intervals, effect sizes, degrees of freedom and *P* value noted<br>*Give P values as exact values whenever suitable.*
☒	☐	For Bayesian analysis, information on the choice of priors and Markov chain Monte Carlo settings
☒	☐	For hierarchical and complex designs, identification of the appropriate level for tests and full reporting of outcomes
☒	☐	Estimates of effect sizes (e.g. Cohen's *d*, Pearson's *r*), indicating how they were calculated

Our web collection on statistics for biologists contains articles on many of the points above.

Software and code

Policy information about availability of computer code

Data collection	Images were classified by volunteers and scientists online using a dedicated project on the Zooniverse platform (https://www.zooniverse.org/projects/gtitcomb/parasite-safari). Identifications were aggregated and checked for misclassifications using R Studio version 4.0.1
Data analysis	The final dataset was analyzed in R Studio version 4.0.1, using the glmmTMB package (v 1.1.2) to fit zero-inflated hurdle generalized linear mixed models. Code for data analysis is provided with data at and X:

For manuscripts utilizing custom algorithms or software that are central to the research but not yet described in published literature, software must be made available to editors and reviewers. We strongly encourage code deposition in a community repository (e.g. GitHub). See the Nature Research guidelines for submitting code & software for further information.

Data

Policy information about availability of data

All manuscripts must include a data availability statement. This statement should provide the following information, where applicable:

- Accession codes, unique identifiers, or web links for publicly available datasets
- A list of figures that have associated raw data
- A description of any restrictions on data availability

All camera trapping, dung density, and parasite density data generated in this study have been deposited in the Environmental Data Initiative database, available publicly at: https://doi.org/10.6073/pasta/479a35c413c9b70cd77d3d88478d0c78. The data package also includes additional source data files for figures.

Field-specific reporting

Please select the one below that is the best fit for your research. If you are not sure, read the appropriate sections before making your selection.

☐ Life sciences ☐ Behavioural & social sciences ☒ Ecological, evolutionary & environmental sciences

For a reference copy of the document with all sections, see nature.com/documents/nr-reporting-summary-flat.pdf

Ecological, evolutionary & environmental sciences study design

All studies must disclose on these points even when the disclosure is negative.

Study description	Data were collected from two research systems: 1) An experimental system consisting of 5 pairs of water pans and a matched matrix site (Ol Pejeta Conservancy), and 2) An observational system consisting of 20 man-made dams and matched matrix sites (Mpala Research Centre and Ol Pejeta Conservancy). At each water source and matrix site, we conducted dung counts in 1m2 quadrats at 10m invervals along six 150m transects that radiated outwardly from water or the center of the matrix site. We resampled transects at experimental sites every ~3 months (n=9 total surveys), and every ~6 months at observational sites (n=5 total surveys for sites at Mpala Research Centre, n=2 surveys at Ol Pejeta). At experimental sites, one survey was conducted at all sites before one pan was drained for a 1-year period (n=5 surveys during this period) before being refilled (n=3 surveys after refilling).<br>Soils were sampled for parasite eggs at each water source and matrix site during each resurvey. Specifically, a 25cm2 area of surface soil (<2cm deep) was sampled from each of the 6 transects at the 0m and 50m mark at water, and at the 50m mark at matrix sites.<br>Camera traps were deployed at all experimental sites (n=15; one at each water pan, one at each matrix site) for the duration of the experiment (September 2016 - September 2018). Camera traps were deployed for one month at observational dams and matrix sites at varying times between April and September 2017. Although all cameras at observational sites did not run concurrently, paired sites (i.e. a dam and its paired matrix) were sampled at the same time.
Research sample	For dung surveys, a research sample consisted of mean dung density (cm3/m2) for each 10m distance interval along transects (averaged over the six transects). For surveys conducted after the 'pre' period at Ol Pejeta, 1m2 quadrats were placed on both sides of transects to increase sampling area. Quadrat area was chosen to enable precise dung measurements and identifications at a scale and spacing interval that allowed us to capture variation in density along the 150m sampling distance radiating from water.<br>For parasites in soil, a research sample was the count of parasite eggs found in each 25g soil aggregate created from subsamples from transects. This sample was chosen to enable efficient laboratory methods to wash, centrifuge, and perform flotation steps in 50mL and 15mL tubes. Identified eggs were of several different parasitic nematode species known to infect herbivores via fecal-oral transmission. A summary of such strongyle and trichostrongyle-type species is provided in SI Appendix Table S2 and S3.<br>For camera trap analyses, a sample was the daily activity (in units of individuals x duration (s)) for each animal of interest. This sample was chosen to minimize the effect of variation in activity within a day.
Sampling strategy	Experimental System: Sample sizes at experimental sites were chosen in coordination with ranching operations at Ol Pejeta to maximize the number of replicates without disturbing cattle ranching operations or greatly reducing water supply; managers allowed a maximum of five pans that could be drained, which therefore determined our sample size in terms of independent locations. Resurveys were conducted every 3 months to capture variation over dry and wet seasons while the water pans were drained. These sample sizes were sufficient in that we were able to detect highly significant effects over the course of the experiment.<br>Observational System: While we did not calculate sample sizes specific to the mixed effects tests conducted in this study, we determined that a site-level sample size of 20 would be sufficient for detecting moderate effect sizes (dz = 0.65 with power = 80) for paired comparisons, provided that site-level differences in means of each response variable were approximately normally-distributed using the G*Power program, version 3.1.9.2.
Data collection	Dung surveys were conducted by J. Mantas, with assistance from J. Hulke, and G. Titcomb. A 1m2 quadrat was laid at each 10m interval and total dung was identified to species, aged, and quantified. For consistency, J. Mantas performed all identifications and estimates of dung freshness and volume, while J. Hulke or G. Titcomb recorded data using pen and paper, confirmed identifications, and assisted in crushing or removing dung.<br>Parasites in soil were determined using a sugar floatation method, in which aggregated soil samples were washed with a detergent to disassociate parasite eggs from soil particles, then suspended in a saturated sugar solution to separate eggs from soil. Eggs that floated to a coverslip were quantified manually using a light microscope. J. Hulke and G. Titcomb performed and recorded all parasite counts in csv files.<br>Cameras were deployed and maintained by G. Titcomb, J. Hulke, and J. Mantas. G. Titcomb and J. Hulke performed preliminary wildlife identifications, and G. Titcomb established the online citizen science site (https://www.zooniverse.org/projects/gtitcomb/parasite-safari) where volunteers conducted additional identifications.
Timing and spatial scale	Experimental System: Surveys were performed along 150m transects before draining water from each experimental pan in October 2016. We repeated dung surveys at each pan and matrix site (every 3 months, n = 5 resurveys during the experiment) before refilling in January 2018 and resurveying until September 2018 (n = 3 surveys post refill). Surveys were conducted at 3-month intervals to capture variation over the two dry seasons and two wet seasons when pans were drained.<br>Observational System: Five surveys were conducted from November 2015 – October 2017 at all sites at Mpala; two surveys were repeated at the Ol Pejeta Dams during November 2015 and September 2016.<br>Total survey area for each site (matrix or water source) spanned approximately 7ha, while the range of experimental sites at Ol Pejeta spanned a ~72km2 area (determined from the convex hull of site coordinates). The range of observational sites at Mpala and Ol Pejeta spanned a ~95km2 and ~8km2 area respectively.
Data exclusions	No collected data were excluded from analyses, with the exception of any camera trap classifications that were filtered due to mis-

identifications or unlikely responses (e.g. number of species present > 7).

Reproducibility	Due to the large size and duration of the experiment, the experiment itself was not repeated. However, we conducted 9 total surveys at the experimental sites to investigate consistency in the results, including these resurveys as effects in our models. In all models, the random effect of 'period' was significant across the duration of the experiment, as this was also related to the experimental status. However, a test of the interaction between treatment and period for egg density for the "During" portion of the experiment (n=5 resurveys) was insignificant (p = 0.28), indicating that treatment effects were consistent across sampling periods. A plot of this relationship showed parallel lines for all treatments across these 5 periods.
Randomization	Matrix site coordinates were randomly selected from a range of pre-determined locations 1km from the experimental water pan or dam and at least 1km from any other water source. Specifically, we used a random number generator (1-360) to determine the angle from which to draw the 1km line. If the resulting location fell within 1km of another water source, outside property borders, or did not match elevation (on/off escarpment), then we re-ran the random number generator. For each experimental pair, we determined the water source to be drained by flipping a coin for 4 out of the 5 pairs. For the remaining pair, only one water source could be drained due to pump configuration (site ID = "Tangi").
Blinding	Blinding was not possible for fieldwork.<br>For camera trap identifications, volunteers did not know the location or experimental status of each site in an image, nor did they know the experimental period (i.e. whether the images were taken prior to, during, or after water manipulation).

Did the study involve field work? ☒ Yes ☐ No

Field work, collection and transport

Field conditions	Field work was conducted from approximately 8am - 4pm at sampling intervals throughout the year. Surveys were not conducted during or immediately following periods of heavy rainfall. Surveys were conducted in temperatures ranging from approximately 20-28 degrees C.
Location	The observational component of the study at 20 man-made dams and paired matrix sites was conducted at Mpala Research Centre (0.283° N, 37.867° E) and Ol Pejeta Conservancy (0.0043° S, 36.9637° E), located in Laikipia County, central Kenya. The experimental component of the study was conducted at Ol Pejeta Conservancy.
Access & import/export	Fieldwork was permitted under the Kenyan National Commission for Science, Technology, and Innovation (NACOSTI/P/16/0782/10585) and Kenya Wildlife Service (KWS/BRM/5001). No samples were exported as part of this study.
Disturbance	Disturbance was minimized by conducting surveys at broad intervals (>3 months), and by avoiding direct animal handling. Cameras were installed on pre-existing structures (e.g. trees), rather than camera poles. Fieldwork was paused in the event that animals came to water. Drained water sources were located near (<1km) permanent water sources, and the experiment was limited to 5 total drained pans to avoid large impacts on cattle ranching and wildlife access to water.

Reporting for specific materials, systems and methods

We require information from authors about some types of materials, experimental systems and methods used in many studies. Here, indicate whether each material, system or method listed is relevant to your study. If you are not sure if a list item applies to your research, read the appropriate section before selecting a response.

Materials & experimental systems

n/a	Involved in the study
☒	☐ Antibodies
☒	☐ Eukaryotic cell lines
☒	☐ Palaeontology and archaeology
☒	☐ Animals and other organisms
☒	☐ Human research participants
☒	☐ Clinical data
☒	☐ Dual use research of concern

Methods

n/a	Involved in the study
☒	☐ ChIP-seq
☒	☐ Flow cytometry
☒	☐ MRI-based neuroimaging
